# Global herpes zoster burden in adults with COPD: a systematic review and meta-analysis

**DOI:** 10.1183/16000617.0167-2025

**Published:** 2026-02-04

**Authors:** Alvaro A. Cruz, Kevin J. Mortimer, Ingrid T. Sepúlveda-Pachón, Hilde Vroling, Charles Williams

**Affiliations:** 1 ProAR Foundation and Federal University of Bahia, Salvador, Brazil; 2 Cambridge Africa, Department of Pathology, University of Cambridge, Cambridge, UK; 3 Department of Paediatrics and Child Health, School of Clinical Medicine, College of Health Sciences, University of KwaZulu Natal, Durban, South Africa; 4 Respiratory Medicine, Aintree University Hospital, Liverpool, UK; 5 P95 Epidemiology and Pharmacovigilance, Bogotá, Colombia; 6 Pallas Health Research and Consultancy, a P95 company, Rotterdam, The Netherlands; 7 GSK, Wavre, Belgium

## Abstract

**Background:**

COPD is associated with an increased risk of infections, such as herpes zoster, potentially leading to greater morbidity and mortality. This systematic review assessed the evidence on herpes zoster burden in COPD.

**Methods:**

A global systematic literature review and meta-analysis was conducted (MEDLINE/Embase, 2003–2024) on herpes zoster burden (incidence, risk, complications, impact on COPD and healthcare resources) in adults aged ≥18 years with COPD.

**Results:**

22 studies on herpes zoster burden in COPD were included. The pooled herpes zoster incidence rate per 1000 person-years in adults with COPD aged ≥18 years was 10.98 (95% CI 8.28–14.56), increasing to 13.95 (10.80–18.02) in adults aged ≥50 years. The pooled risk ratio of developing herpes zoster was 1.49 (1.17–1.89) in adults aged ≥18 years with COPD and 1.86 (1.28–2.69) in COPD treated with corticosteroids. The pooled rate ratio of developing post-herpetic neuralgia (persistent pain lasting ≥90 days) was 1.50 (1.10–2.04) in adults with herpes zoster and COPD *versus* with herpes zoster alone. Herpes zoster was linked to higher healthcare costs and resource use, and may be associated with COPD exacerbations. Study designs, settings, case definitions, sample sizes and study periods differed, resulting in heterogeneity.

**Conclusions:**

Adults with COPD have an increased risk of herpes zoster and complications and an associated burden on healthcare systems, with higher risks in those on corticosteroids. Herpes zoster vaccines offer effective protection, including for adults with COPD, and could help reduce the disease and its economic burden.

## Shareable abstract

**A review and meta-analysis found herpes zoster and complications risk rose in adults with COPD, especially those treated with corticosteroids. Herpes zoster increased healthcare resource use and costs in COPD patients; vaccination could reduce this burden.** https://bit.ly/4qZBdg5

## Introduction

COPD is a common chronic respiratory disease in adults (particularly over the age of 40–50 years) [1], with symptoms of dyspnoea, chronic cough, sputum production and risk of acute exacerbations. Worldwide in 2019, there were 212.3 million people with COPD, with the highest prevalence in South and East Asia and Western Europe [2], and 3.3 million deaths due to COPD. COPD is a leading cause of morbidity and the third commonest cause of mortality. It is also associated with other comorbidities, such as cardiovascular disease, diabetes, lung cancer, and other respiratory infections [1, 3]. The economic burden on healthcare systems is substantial, costing EUR 38.6 billion per year in the European Union alone, and accounting for an estimated 56% of all respiratory healthcare costs [1]. Goals of treatment, described by the Global Initiative for Chronic Obstructive Lung Disease (GOLD) [1], include reducing symptoms and acute exacerbations. COPD is associated with immune dysfunction and a higher risk of infections due to chronic lung inflammation [4] and corticosteroid treatments [5]. Infections can increase morbidity and mortality, and therefore, preventive strategies are recommended (*e.g.* coronavirus disease 2019, influenza, pneumococcal, pertussis and herpes zoster vaccination, and respiratory syncytial virus vaccination) [1].

Herpes zoster is a common and painful disease that typically occurs in adults, and can significantly impact quality of life, affecting approximately a third of the general population over the course of their lifetime. The risk of herpes zoster increases with age, with higher incidence rates in older age groups (*e.g.* 5.15/1000 population in ages 50–54 years *versus* 11.27/1000 in ages ≥85 years [6]) [7–9]. Up to 30% of people with herpes zoster develop long-term chronic nerve pain (post-herpetic neuralgia (PHN)) that can last from months to years [10], and ∼10% develop other non-PHN complications (*e.g.* herpes zoster ophthalmicus (HZO) with ocular involvement, stroke, Ramsay Hunt syndrome), which occur more frequently in high-risk populations. Herpes zoster is caused by reactivation of the latent varicella zoster virus (VZV), which is present in >90% of adults, placing them at risk of herpes zoster [11]. Reactivation of VZV is thought to be driven by impaired cell-mediated immunity due to ageing, underlying diseases and/or immunosuppressive therapies [9, 12, 13].

Previous systematic reviews on potential herpes zoster risk factors [14–16] found that adults with COPD have a ∼31–55% increased risk of developing herpes zoster *versus* the general population. A recent systematic literature review focused on herpes zoster in adults with asthma [17]. To the best of our knowledge this is the first systematic literature review focused specifically on herpes zoster in adults with COPD. The objective was to compile the current evidence on the global burden of herpes zoster in adults with COPD, including incidence, morbidity and mortality, PHN and other complications, COPD as a risk factor for herpes zoster, and impact on underlying disease and healthcare resource use (HCRU).

## Methods

A systematic literature review was conducted according to the Cochrane [18] and Preferred Reporting Items for Systematic Review and Meta-Analyses [19] guidelines for performing and reporting systematic reviews. The review is registered in the PROSPERO database (registration number CRD 42024513214).

### Search strategy

Disease-specific terms for herpes zoster were combined with COPD terms in searches in MEDLINE (accessed *via* PubMed) and Embase databases (supplementary file S1). To avoid restricting the search, no outcome-specific terms were included. Publications were included from any country, published in English, French, German, Spanish and Italian, from 1 January 2003, until the search date of 17 February 2024.

### Screening and selection

Records identified from the searches were imported and deduplicated in Rayyan [20]. The process of selection and inclusion/exclusion of articles was registered in an EndNote library by one researcher.

All titles and abstracts were independently screened by two researchers (I.T. Sepúlveda-Pachón and H. Vroling); 30% of full-text articles selected by these two researchers were compared and reviewed for selection relevancy. Reasons for exclusion of full-text articles are provided in supplementary file S2. Hand-searching of reference lists was performed from identified systematic literature reviews to check for potentially missed studies meeting the selection criteria.

Studies were selected based on the population, outcomes and study design criteria. The population of interest was adults aged ≥18 years with COPD. Outcomes of interest were incidence/prevalence, risk of herpes zoster and its complications (*i.e.* PHN, disseminated zoster, encephalitis, ophthalmic zoster, or pneumonia), hospitalisation, mortality, exacerbation/worsening of COPD due to herpes zoster, and HCRU costs. Study designs included were observational studies and phase 3 clinical trials. Other study designs were excluded, as well as small studies (sample size of <30 participants), and publication types such as letters to the editor, editorials, conference abstracts and comments.

### Data extraction

Key data were extracted into standardised tables in Microsoft Excel, including citation; study methods (design, study period, setting and country); population (inclusion/exclusion criteria, size, age group, gender, type of patients (inpatients/outpatients)); follow-up time, denominator; herpes zoster case detection, herpes zoster and PHN case definition; COPD case definition, severity, duration of disease, treatments; outcomes (by year, gender, age and treatment); comments or quality issues from authors as well as researchers. If relevant data were only presented in a figure, the authors were contacted to request the full data. Data extraction was performed by one researcher, and 30% of extracted data were checked by a second researcher.

### Quality assessment

The checklists developed by the Joanna Briggs Institute (University of Adelaide, Adelaide, Australia) for cohort studies, case–control studies, cross-sectional and incidence/prevalence studies were used for the methodological quality assessment of all full-text articles included (supplementary file S2).

### Data synthesis

Data were summarised descriptively by outcome and, where feasible, by age and medication use. The following Cochrane criteria [18] were used for the feasibility assessment of conducting a meta-analysis: 1) all outcomes are comparable and can be pooled meaningfully; 2) the correct data are available for the included studies. For outcomes reported as proportions or rates (*e.g.* incidence per 1000 person-years) either the numerator or the denominator value needs to be available to be able to pool results; 3) all interventions and comparators are the same, or similar enough to be combined meaningfully. For observational studies, the assessment of this criterion focuses on the comparability of study characteristics (*i.e.* setting, period, definitions of herpes zoster and of COPD) and population characteristics (*i.e.* age, gender, medication). A minimum of three studies was considered sufficient for a meta-analysis, provided they could be meaningfully pooled, and their results were sufficiently similar.

### Meta-analysis methods

A random effects meta-analysis was used to summarise the following outcomes that could be pooled according to the feasibility assessment: 1) the incidence of herpes zoster in adults with COPD; 2) COPD as a risk factor for developing herpes zoster; and 3) COPD as a risk factor for developing PHN. A random-effects model was chosen, as characteristics of study design, population, and setting differed between studies.

For COPD as a risk factor for herpes zoster and PHN, the risk ratio was the preferred metric. Nested case–control studies reporting odds ratios were included in the meta-analysis because odds ratios in nested case–control studies estimate the risk ratio [21, 22]. Other case–control studies reporting odds ratios based on incident herpes zoster, conducted in dynamic and stable populations, were also included in the meta-analysis, as these odds ratios also provide an estimate of risk ratio [22–24]. Studies reporting hazard ratios (HR) and relative risks were also included in the meta-analysis. The odds ratio results from classical case–control studies were not included, as these can only be interpreted as risk ratios in cases of rare disease (typically if risk <10%) [25], which is not the case for herpes zoster.

In case a study only reported stratified incidence estimates, an overall incidence was calculated using the reported numerator and/or denominator, if available.

A restricted maximum-likelihood (REML) and Paule–Mandel (PM) random-effects model was used for the aggregation of incidence data and rate ratio results, respectively, while using a generalised linear mixed model approach for the prevalence aggregation. The level of heterogeneity was assessed using Cochran's Q statistics and I^2^. Forest plots were used to present the pooled estimates with 95% confidence intervals and prediction intervals, and to explore age as a source of heterogeneity in subgroup analyses, if feasible. In the subgroup analysis, the pooled estimate in each subgroup and within-group heterogeneity were obtained. To test the robustness of results, a pre-specified sensitivity analysis was conducted allowing a broader inclusion of studies in the meta-analyses. In the case of a small number of studies (fewer than five), a *post hoc* sensitivity analysis using the Hartung–Knapp–Sidik–Jonkman (HKSJ) method was performed to test the robustness of the results obtained through the REML/PM method. Publication bias was assessed if ≥10 studies were included in each analysis, using funnel plots and the Egger test. Data analysis was performed using R version 4.3.1 (R Core Team 2018, Vienna, Austria).

## Results

**FIGURE 1:**
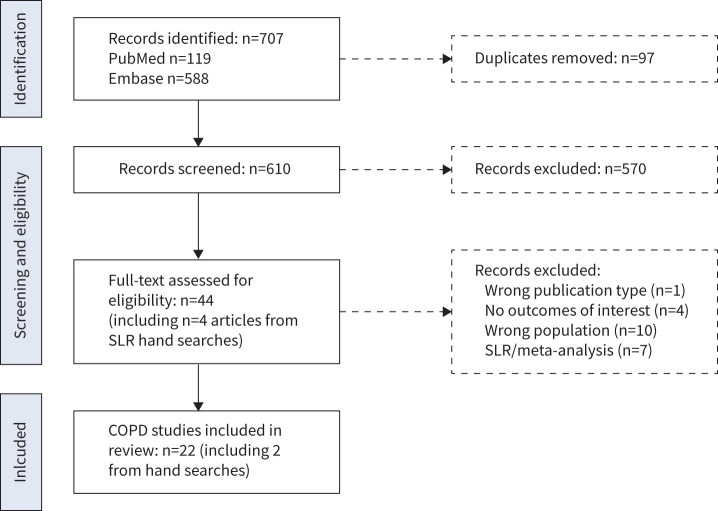
Preferred Reporting Items for Systematic Review and Meta-Analyses flowchart showing the results of the search and selection process, with 22 studies included for COPD. SLR: systematic literature review.

The search identified 610 unique records from MEDLINE and Embase, and an additional four from hand searches; overall, 44 underwent full-text screening. Rayyan identified and removed all duplicates prior to screening. There were 22 studies included on herpes zoster in adults with COPD (figure 1). A list of excluded studies with reasons for exclusion can be found in supplementary file S3.

### Study characteristics

The study characteristics of the 22 included studies are summarised in supplementary table S1.

The majority of the studies were conducted in Europe (n=12), including Spain [26–30], the United Kingdom [31, 32], Denmark [33], France [34], Germany [35], Italy [36] and Sweden [37]. Seven studies were from North America, including the United States of America (USA) [38–43] and Canada [44]; and three were from Asia, including Japan [45, 46] and Taiwan [47].

Most studies were comparative observational studies, using either a case–control design (n=6 [30, 31, 33, 34, 39, 40]; five were matched [31, 33, 34, 39, 40]) or cohort design (n=9 [28, 35, 36, 38, 41, 42, 45–47]; two were matched [35, 47]). In addition, there were two cross–sectional studies [43, 44] and five retrospective surveillance studies [26, 27, 29, 32, 37].

The International Classification of Diseases (ICD-9/10) or equivalent codes were used to define herpes zoster, except in three studies [30, 34, 43] (one used a general practitioner diagnosis [34], one used patient self-report [43] and one did not describe how herpes zoster was defined [30]). Studies defined COPD cases using ICD or equivalent codes (some also required prescriptions for COPD medication [36, 38, 42, 47]), except for five studies: one used extensive clinical criteria [44], one used patient self-reported physician diagnosis [43], and three did not report how COPD cases were defined [30, 34, 40].

Most studies reported on COPD as a risk factor for herpes zoster (n=16) [26, 28–35, 39–42, 44, 46, 47] and herpes zoster incidence rates (n=12) [26, 28, 31, 35–37, 41, 42, 44–47], while herpes zoster complications (PHN [29, 30, 32, 37, 42, 43], HZO [43] and vascular complications [38]) were reported in seven studies, and three studies reported on herpes zoster risk by corticosteroid use [35], inhaled corticosteroid (ICS) [28], and ICS or oral corticosteroid (OCS) use [47]). Economic outcomes (*i.e.* costs of herpes zoster in COPD [27, 36, 38]) and worsening of COPD due to herpes zoster [28, 38, 43] were reported in three studies each, and one study reported on mortality in herpes zoster patients with COPD [27].

The quality of the studies was assessed (supplementary file S3). Among six case–control studies, two were of high quality [31, 40]. Lower quality assessments were related to an unclear diagnosis of COPD [30, 34, 40], the herpes zoster definition used [33], and limited statistical analysis [30, 34] or exposure period definition [39]. Among the nine cohort studies, none specified whether follow-up was complete or provided reasons for loss to follow-up. Lower quality also related to definitions of COPD [36, 38, 42, 47] and herpes zoster [35, 36, 45–47] used, limitations in statistical analyses [28, 36, 38, 45]. Both cross-sectional studies failed to report inclusion criteria clearly and had analysis limitations [43, 44]. One of the five surveillance studies was of high quality [26], while the others had issues reporting potential incomplete follow-up mitigation strategies [29, 32] and population data [27, 37].

### Herpes zoster incidence in adults with COPD

Eight studies reported herpes zoster incidence rates per 1000 person-years [26, 31, 35, 41, 42, 45–47], and four reported cumulative incidence per 1000 population [28, 36, 37, 44] (supplementary table S2).

Herpes zoster incidence rates varied from 13.0 (age ≥40 years) to 17.7 (age ≥65 years) per 1000 person-years [41, 42, 47], with lower rates in populations including younger adults (8.7–11.4 in adults aged ≥18 years [26, 35] or 2.3 in adults aged ≤50 years [31]). Incidence generally increased with age [31, 42, 45–47]. The cumulative incidence range was broader (6.9–12.3/1000 population aged ≥18 years [37, 44]) and also increased with age [28, 37]. In Germany, herpes zoster incidence rates in adults (aged ≥18 years) with COPD increased slightly between 2008 (8.2/1000 person-years) and 2018 (8.7/1000 person-years) [35]. Herpes zoster incidence tended to be higher in women than in men in Spain [26, 28], Japan [45, 46] and Canada [44]. Herpes zoster incidence was higher in adults (aged ≥50 years) with COPD using OCS (26.3/1000 person-years), followed by ICS (18.4/1000 person-years) *versus* no corticosteroids (14.3/1000 person-years) [47]; or in adults using ICS (13.0/1000 population) *versus* no ICS (11.1/1000 population) [28].

**FIGURE 2:**
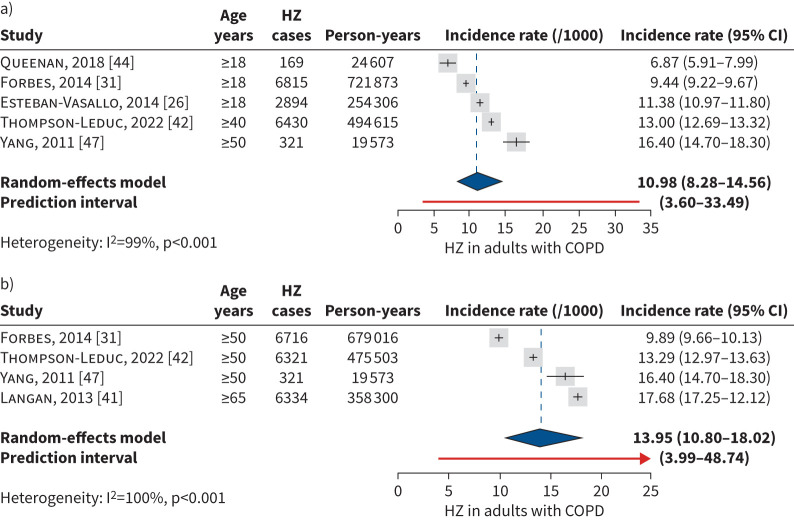
Meta-analysis of herpes zoster (HZ) incidence (per 1000 person-years) in adults with COPD: a) overall population aged ≥18 years; and b) population aged ≥50 years. In the overall population analysis, studies in adults aged ≥18 years were likely to include an age group aged ≥40/50 years, due to COPD epidemiology.

Overall, six studies had sufficiently similar characteristics to conduct a meta-analysis for herpes zoster incidence rate. Five studies were included in an overall incidence rate analysis for adults with COPD (aged ≥18 years). As COPD is rarely diagnosed before the age of 40–50 years [1, 48], this analysis included three studies in adults aged ≥18 years [26, 31, 44], one in adults aged ≥40 years [42], and one in adults aged ≥50 years [47]. One study, which reported cumulative incidence per 1000 population [44], was included as the study period was 1 year and no loss to follow-up was assumed. Two studies were excluded due to missing numerators and denominators [35, 46]; and one due to exclusion of adults aged >74 years [45]. The pooled herpes zoster incidence rate per 1000 person-years in adults aged ≥18 years with COPD was 10.98 (95% CI 8.28–14.56) (figure 2a). As herpes zoster incidence is known to increase with age, four studies were included in a meta-analysis of herpes zoster incidence rate in adults with COPD aged ≥50 years [31, 41, 42, 47], although one study included a population aged ≥65 years [41]. The pooled herpes zoster incidence rate per 1000 person-years in adults with COPD aged ≥50 years was 13.95 (95% CI 10.80–18.02) (figure 2b).

When stratifying studies by further age groups, the pooled incidence rate (per 1000 person-years) of herpes zoster in adults with COPD aged 50–59 years was 9.69 (95% CI 5.49–17.10), increasing to 11.97 (8.83–16.23) in adults aged 60–69 years and to 14.90 (12.21–18.18) in adults aged ≥70 years (supplementary figure S1).

In the *post hoc* sensitivity analysis using the HKSJ method, the estimates were similar but had slightly wider confidence intervals.

### Risk of herpes zoster in adults with COPD

Of the 14 studies [26, 28, 30, 31, 33–35, 39–42, 44, 46, 47] that investigated the risk of herpes zoster in adults with COPD *versus* no COPD (supplementary table S3), all but one study (in France [34]) showed an increased risk.

Compared with controls without COPD, COPD was a significant risk factor for developing herpes zoster, with adjusted (a)ORs of 1.10–1.22 in adults aged ≥18 years [30, 31, 35] and aOR of 1.35 in 18–64-year-olds [39], an adjusted (a)HR of 1.24 in 18–74-year-olds [46], and an adjusted risk ratio of 1.83 *versus* controls without specific chronic diseases [44], (all 95% CI lower limits were >1). In older adult age groups, the risk of developing herpes zoster remained significant: in adults aged ≥40 years, aOR 1.20 [33]; in adults aged ≥50 years, adjusted risk ratio 1.45 [28] and aHR 1.68 [47]; and in adults aged ≥65 years, aHR 1.17 [41]. In studies presenting age-stratified risks [31, 35, 42, 47], COPD was a significant risk factor for herpes zoster in all age groups, and an increased risk among older age groups was reported in two studies [31, 42]. COPD was reported to be a significant risk factor for herpes zoster diagnosed in the hospital (aOR 2.05) [33]. Only one study stratified herpes zoster risk by sex and no difference in risk was found [26]. Two studies with low numbers of participants with COPD (n=41, both studies) reported a nonsignificant increased risk (OR 1.48, 95% CI 0.77–2.84) [40], and a reduced risk of herpes zoster (OR 0.63, 95% CI 0.29–1.23) in a study with COPD diagnosis based on self-report [34], which might have led to COPD status not being accurately classified.

When assessing herpes zoster risk by COPD treatment, the risk increased from adjusted risk ratio 1.45 (overall) to 1.61 (treated with ICS) in Spain [28]; and from aHR 1.67 (no corticosteroids) to aHR 2.09 (treated with ICS) and aHR 3.00 (treated with OCS) in Taiwan [47], in adults aged ≥50 years. In Germany, adults of all ages with COPD and treated with systemic corticosteroids had a significant risk of developing herpes zoster (aOR 1.25–1.54), while among individuals without systemic corticosteroids, herpes zoster risk was significant only for adults aged <50 years (aOR 1.23) [35].

Eight studies [28, 31, 33, 35, 40–42, 47] had sufficiently similar characteristics to conduct a meta-analysis for COPD as a risk factor for herpes zoster in adults (one study [41] included for analysis of ages ≥50 years was not eligible for the ≥18-years analysis). Similar to the incidence rate meta-analysis, as COPD is rarely diagnosed before the age of 40–50 years [1, 48], studies focusing on adults aged ≥18 years were grouped with studies in adults aged ≥40–50 years. For the study in Germany with risk data by year (2008–2018), the mid-period values (from 2013) were used, as no overall incidence was reported, and incidence increased slightly over time [35]. Six studies were excluded from the meta-analysis: case–control studies in which the OR cannot be converted to an risk ratio [34, 39]; a study only presenting sex-stratified data [26]; a study in a population aged 18–74 years [46] and a study in a population aged ≥65 years that could not be meaningfully combined with other studies [41]; and a study lacking sufficient details on population and follow-up time to permit similarity assessment [30].

**FIGURE 3:**
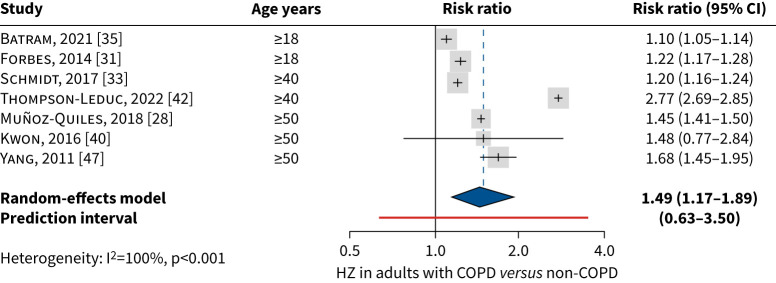
Meta-analysis of COPD (*versus* non-COPD) as a risk factor for herpes zoster in adults aged ≥18 years. HZ: herpes zoster.

In the overall analysis, the pooled risk ratio of developing herpes zoster was 1.49 (95% CI 1.17–1.89) in adults aged ≥18 years with COPD (figure 3). Heterogeneity was high (100%). The significant association between COPD and herpes zoster remained (pooled risk ratio 1.39, 95% CI 1.16–1.65) in the pre-specified sensitivity analysis with broader inclusion of articles, including the Japanese population aged 18–74 years [46], the USA population aged ≥65 years [41], and the Spanish study with insufficient reporting of relevant methodology aspects [30] (supplementary figure S2a1). There was no evidence of publication bias (Egger's test p=0.873) (supplementary figure S2a2).

Separate meta-analyses were conducted for studies with adults aged ≥50 years [28, 31, 35, 40, 41, 47]; and in subgroups with age-stratified data for adults aged 50–59 years [31, 35, 42, 47], 60–69 years [31, 42, 47] and ≥70 years [31, 41, 47]. COPD remained significantly associated with herpes zoster in each age group assessed, with a pooled risk ratio 1.31 (95% CI 1.12–1.52) in ages ≥50 years; pooled risk ratio 1.61 (95% CI 1.04–2.49) in 50–59-year-olds; pooled risk ratio 1.98 (95% CI 1.33–2.94) in 60–69-year-olds; and pooled risk ratio 1.36 (95% CI 1.07–1.72) in ≥70-year-olds, with no significant difference in risk between age groups (supplementary figure S2b and c). In a *post hoc* sensitivity analysis using the HKSJ method, herpes zoster risk was higher in adults in each subgroup, but with wider confidence intervals (nonsignificant).

**FIGURE 4:**
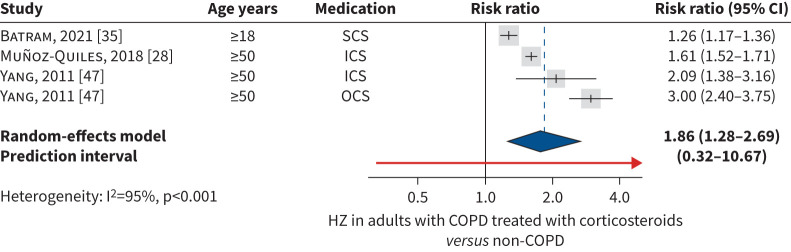
Meta-analysis of COPD treated with corticosteroids (*versus* non-COPD) as a risk factor for herpes zoster (HZ). Studies in adults aged ≥18 years were likely to include an age group aged ≥40 years, due to COPD epidemiology. SCS: systemic corticosteroids; ICS: inhaled corticosteroids; OCS: oral corticosteroids.

To assess the impact of corticosteroid use, data from three studies were pooled on the risk of herpes zoster in adults with COPD treated with corticosteroids *versus* adults without COPD [28, 35, 47]. The studies included corticosteroids administered systemically [35], ICS [28], and ICS or OCS [47]. The pooled risk ratio of developing herpes zoster in COPD treated with corticosteroids was 1.86 (95% CI 1.28–2.69) (figure 4). In a *post hoc* sensitivity analysis using the HKSJ method, herpes zoster risk remained significantly higher in adults with COPD treated with corticosteroids.

### Herpes zoster complications and mortality in adults with COPD

One study assessed the incidence of PHN in adults aged ≥40 years, using person-years from herpes zoster onset as the denominator [42]. The overall incidence was estimated at 64.8 per 1000 person-years, increasing from 29.9 person-years (aged 40–49 years) to 76.7 person-years (aged ≥80 years).

Five studies [29, 30, 32, 37, 43] reported the proportion of adults with COPD and herpes zoster who developed PHN. For PHN defined as pain persisting for ≥90 days following herpes zoster onset, results varied from 13.1% to 26% [29, 32, 37, 43], with the higher proportion reported in a subgroup with PHN defined based on PHN ICD codes or herpes zoster ICD codes plus neuropathic pain prescriptions. PHN proportions varied when stratified by duration of PHN, *i.e.* 29.6% (30–180 days), 21.5% (90–180 days) and 14.4% (150–180 days) [29]. When stratified by age, higher PHN (≥90 days) proportions were reported in older (14.1% in those aged ≥60 years, 15.0% in those aged ≥70 years) *versus* younger adults (5.8% in those aged 18–60 years) [32]. A meta-analysis was feasible for three studies in adults aged ≥50 years [29, 32, 43]. Two studies were excluded, due to lack of details on numerator and denominator [37], and on herpes zoster, PHN and COPD definitions [30]. The pooled proportion of adults aged ≥50 years with COPD and herpes zoster who developed PHN was estimated at 16.4% (95% CI 12.5–21.2%). In the pre-specified sensitivity analysis, including the Spanish study with limited population definitions [30], the pooled proportion of PHN was 15.7% (95% CI 12.6–19.3%).

Four studies [29, 30, 32, 42] reported on COPD as a risk factor for PHN. COPD was a significant risk factor for PHN: in adults aged ≥18 years (aOR 1.24–1.53) [30, 32]; and in adults ≥50 years (adjusted risk ratio 1.86) [29]. One study reported a nonsignificant increased risk of PHN (adjusted incidence rate ratio (aIRR) 1.07, 95% CI 0.79–1.45) after adjusting the model (*versus* unadjusted IRR 1.75, 95% CI 1.17–2.62) [42]. Data from three studies on COPD as a risk factor for PHN were pooled, which defined PHN within a timeframe of ≥90 days after herpes zoster [29, 32, 42]. The studies included populations aged ≥18, ≥40 and ≥50 years, as all adjusted for age. One study was excluded from the meta-analysis as it provided insufficient details to assess similarity, and the OR could not be converted to a risk ratio in the context of an unmatched case–control study where the outcome (PHN) is not rare [30].

**FIGURE 5:**
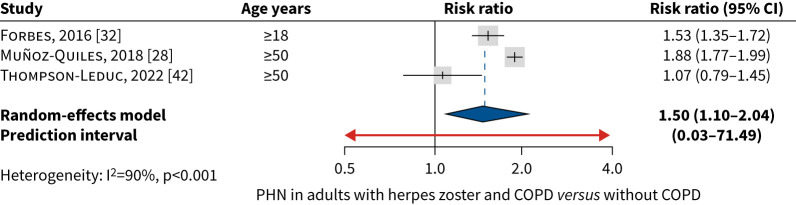
Meta-analysis of post-herpetic neuralgia (PHN) risk in adults with herpes zoster and COPD (*versus* without COPD). Studies in adults aged ≥18 years were likely to include an age group aged ≥40 years, due to COPD epidemiology.

The pooled rate ratio of developing PHN was 1.50 (95% CI 1.10–2.04) in adults with herpes zoster and COPD *versus* adults with herpes zoster without COPD (figure 5). In *post hoc* sensitivity analyses using the HKSJ method, PHN risk remained higher in adults with COPD, but with wider confidence intervals (*i.e.* nonsignificant).

Other herpes zoster complications in adults with COPD were reported in two studies and included HZO and vascular complications. A study from the USA in adults aged ≥50 years with COPD reported that 7.3% of herpes zoster events had HZO complications [43]. Another study from the USA compared adults with COPD and herpes zoster to adults with COPD without herpes zoster, and reported significantly higher incidence rates of stroke (aIRR 1.54, 95% CI 1.18–2.01) and transient ischaemic attack (aIRR 1.39, 95% CI 1.14–1.69) in those with COPD and herpes zoster, with the greatest risk in the first month after herpes zoster. Incidence rates of myocardial infarction were similar in both cohorts (aIRR 0.82, 95% CI 0.61–1.11) [38].

In Spain, a surveillance study in adults with COPD hospitalised with herpes zoster (n=3633) reported inpatient mortality rates of 4.2% (aged 50–59 years), 2.7% (aged 60–69 years) and 5.0% (aged ≥70 years) following herpes zoster [27]. No other studies reported on herpes zoster mortality.

### Exacerbation/worsening of COPD

Three studies [28, 38, 43] reported on exacerbations or worsening of COPD in adults aged ≥50 years with herpes zoster. A survey from the USA in adults aged ≥50 years with COPD and herpes zoster, using self-reported data, stated that during the herpes zoster infection, 23 (12%) experienced a COPD flare/exacerbation and 49 (25.5%) had increased COPD symptoms or dyspnoea [43]. In a retrospective cohort study from the USA, the incidence of COPD exacerbations was increased in the period surrounding herpes zoster onset (*i.e.* during 1 month before to 2 months after herpes zoster onset) compared with the 3 months prior to this period (*post hoc* analysis, IRR 1.13, 95% CI 1.03–1.23), due to a spike in exacerbations in the month before herpes zoster diagnosis. This period appeared to correspond to the prodromal herpes zoster phase before the appearance of the characteristic rash that typically leads to herpes zoster diagnosis [38]. In addition, this study reported significantly higher COPD-related resource use in COPD patients with *versus* without herpes zoster: for any medical service use (aIRR 1.27, 95% CI 1.21–1.34), emergency department visit (aIRR 1.41, 95% CI 1.30–1.53), outpatient visit (aIRR 1.29, 95% CI 1.22–1.35) and other medical services (aIRR 1.26, 95% CI 1.13–1.41) [38]. In Spain, the impact of herpes zoster on COPD was compared in the 6 months before *versus* 6 months after herpes zoster onset, and hospital length of stay (LOS) tended to be longer after herpes zoster (mean ratio 1.19, 95% CI 1.00–1.42); however, the increase was not significant [28].

### Healthcare resource use and costs in adults with COPD and herpes zoster

Five studies [27, 28, 36, 38, 43] reported HCRU in adults aged ≥50 years with COPD and herpes zoster, of which three [27, 36, 38] also reported costs (supplementary table S4). All costs were converted to EUR 2023 (December 2023 values EUR 1=USD 1.0985).

A survey from the USA (n=735) reported that 10.9% of adults with COPD required herpes zoster related hospitalisation and 98.3% required other healthcare services for herpes zoster [43]. The mean LOS for herpes zoster hospitalisation in adults with COPD was 18.4 days in an Italian study (1553 (8.8%) herpes zoster cases hospitalised) [36]; and 15.9 days (age 50–59 years), 14.2 days (age 60–69 years) and 12.9 days (age ≥70 years) in a Spanish study [27]. In a study from the USA, adults with COPD who developed herpes zoster had significantly higher all-cause HCRU than adults with COPD without herpes zoster, including any medical services (aIRR 1.17, 95% CI 1.14–1.21); emergency department visits (aIRR 1.28, 95% CI 1.20–1.35); outpatient visits (aIRR 1.18, 95% CI 1.15–1.22); and other medical services (aIRR 1.13, 95% 1.07–1.19) [38]. A study in Spain found significantly higher herpes zoster related HCRU for adults with herpes zoster and COPD *versus* herpes zoster without COPD, including hospitalisation (OR 2.66, 95% CI 2.17–3.24); outpatient visits (risk ratio 1.05, 95% CI 1.03–1.08); and herpes zoster medication use (risk ratio 1.25, 95% CI 1.19–1.31) [28].

Mean herpes zoster hospitalisation costs for adults aged ≥50 years with COPD were EUR 4248 in Italy [36]; EUR 6584 in the USA [38]; and EUR 6111 (age 50–59 years), EUR 5430 (age 60–69 years) and EUR 5448 (age ≥70 years) in Spain [27]. In USA, the mean±sd total costs (all-cause) per person per month for adults with COPD and herpes zoster in the first year after herpes zoster onset were EUR 3769±6933 and were significantly higher *versus* COPD without herpes zoster (EUR 3413±7241, p<0.004) [38]. The largest cost increase was in the first month after herpes zoster onset [38].

## Discussion

This global systematic literature review and meta-analysis included 22 observational studies describing the burden of herpes zoster in adults with COPD, including herpes zoster incidence, herpes zoster risk and complications, and impact of herpes zoster on COPD exacerbation/worsening and HCRU.

Based on individual study data, herpes zoster incidence in adults with COPD increased with age and corticosteroid use and tended to be higher in women than men. The pooled herpes zoster incidence rate was 10.98 (95% CI 8.28–14.56) in the overall COPD population (aged ≥18 years), and 13.95 (95% CI 10.80–18.02) for adults with COPD aged ≥50 years. Subgroup meta-analysis by age did not confirm a significantly increased herpes zoster incidence with increasing age, but analyses were hampered by large heterogeneity and inclusion of few studies, resulting in large confidence intervals. The risk of herpes zoster in adults with COPD was investigated in 14 studies. The pooled risk ratio of developing herpes zoster was 1.49 (95% CI 1.17–1.89) in the overall COPD population and 1.31 (95% CI 1.12–1.52) in those aged ≥50 years. Results remained consistent and robust in sensitivity analyses. In subgroup analyses in age groups 50–59 years, 60–69 years and ≥70 years, COPD remained a significant risk factor for herpes zoster in all age groups with no significant difference in risk by age group. These results are comparable to the findings from systematic literature reviews on risk factors for herpes zoster, where COPD was associated with a significantly increased risk of herpes zoster (risk ratio 1.31, 95% CI 1.22–1.41) in adults (systematic literature review with studies published in 2003–2017) [15]; risk ratio 1.41 (95% CI 1.28–1.55) in all ages (systematic literature review 1966–2019) [14]; and OR 1.55 (95% CI 1.04–2.31) in all ages (systematic literature review 2003–2022) [16]. The use of corticosteroids appeared to increase the risk of herpes zoster, with a pooled risk ratio in adults with COPD treated with any corticosteroids of 1.86 (95% CI 1.28–2.69) *versus* adults without COPD, which remained significant in *post hoc* sensitivity analysis using the HKSJ method. The findings may reflect the impact of disease severity on risk of herpes zoster, as more severe COPD cases are generally treated with corticosteroids. While the risk of herpes zoster is usually associated with systemic rather than inhaled corticosteroids use [17], an increased risk of herpes zoster was found for systemic, inhaled and oral corticosteroids use in the pooled studies, *versus* controls with no COPD. In one of the studies, herpes zoster incidence in adults aged ≥50 years with COPD was significantly higher in ICS users (13.0, 95% CI 12.3–13.8) *versus* no-ICS users (11.1, 95% CI 10.7–11.4) [28]. Similarly, a recent cohort study from the USA assessing herpes zoster risk in adults with COPD on long-term ICS *versus* short-term (<3 months) or no ICS exposure reported a significantly higher herpes zoster risk in those with longer ICS exposure (adjusted HR 2.57, 95% CI 2.55–2.60) [49].

The risk of herpes zoster complications may also be increased in adults with COPD. The pooled proportion of adults with COPD and herpes zoster who developed PHN was estimated at 16.4% (95% CI 12.5–21.2%), and the pooled risk ratio of developing PHN was 1.50 (95% CI 1.10–2.04) in herpes zoster with COPD *versus* without COPD. In *post hoc* sensitivity analyses using the HKSJ method, PHN risk remained higher in adults with COPD, but with wider confidence intervals (nonsignificant). Our literature search was limited to the most frequent complications of herpes zoster, but some of the retrieved studies mentioned other types of complications. A study from the USA reported significantly higher incidence rates of complications such as stroke (aIRR 1.54, 95% CI 1.18–2.01) and transient ischaemic attack (aIRR 1.39, 95% CI 1.14–1.69) for adults with COPD and herpes zoster *versus* COPD without herpes zoster. Adults with COPD generally have a higher underlying risk of cardiovascular disease [50], and therefore may be more susceptible to herpes zoster-associated vascular events. A recent cohort study from the USA reported that non-herpes zoster vaccinated adults with COPD who developed herpes zoster had a higher risk of stroke (aOR 1.93; p<0.001) and myocardial infarction (aOR 2.61; p<0.001) post-herpes zoster, and that herpes zoster vaccination appeared to reduce the risk by ∼25% [51]. Further studies on the susceptibility of patients with chronic respiratory conditions to herpes zoster-associated vascular complications are warranted.

Herpes zoster may also have an impact on underlying COPD, although data were limited, and assessment measures varied. Three studies reported exacerbations or worsening of COPD in adults aged ≥50 years with herpes zoster; including increased exacerbations and symptoms during herpes zoster [43]; increased exacerbations in the period surrounding herpes zoster onset [38]; and longer hospital LOS in the 6 months after herpes zoster onset [28]. Corticosteroids given during exacerbations may increase herpes zoster risk, potentially affecting the observed outcomes, but do not completely account for differences in COPD-related HCRU after a herpes zoster episode. How herpes zoster may impact COPD is unclear, but may be related to rash location and pain. The most commonly affected dermatome is the thoracic region [52], and acute debilitating pain is the most common symptom of herpes zoster [8, 53]. Pain can influence respiratory rate causing increased ventilation or hyperventilation [54]. Pain is also associated with impaired sleep quality, fatigue and depression, which may add to COPD symptom burden [55]. A recent study explored the association between herpes zoster and subsequent exacerbations [56], reporting that the inflammatory immune response due to herpes zoster could exacerbate chronic inflammation, indirectly leading to increased exacerbations; and found that herpes zoster vaccination was associated with a reduced risk of hospitalisation for COPD exacerbations [56]. This may explain some of the findings reported; however, more research in this area is warranted.

Studies reporting on HCRU and costs found that ∼8.8–10.9% of adults with COPD and herpes zoster required hospitalisation [36, 43], with a mean LOS of 12.9–18.4 days [27, 36]. HCRU was significantly higher in adults with COPD and herpes zoster *versus* COPD alone (regarding any medical service use, emergency department, and outpatient visits [38]); and *versus* herpes zoster alone (regarding hospitalisation, outpatient visits and medication use [28]). Mean hospitalisation costs ranged between EUR 4248 and EUR 6584 in Italy [36] and the USA [38], and emergency department, outpatient costs and total costs were significantly higher *versus* adults with COPD without herpes zoster in the USA [38]. Despite country variations in HCRU and costs, an episode of herpes zoster in adults with COPD consistently placed a significant burden on healthcare systems and increased costs.

COPD is associated with immune dysregulation [47, 57], as well as immunosuppression from corticosteroid use [35, 47], which may put patients at higher risk of developing herpes zoster [47]. The GOLD guidelines currently recommend several vaccinations to prevent infections and help to reduce morbidity and mortality in adults with COPD, including herpes zoster vaccination [1]. The recombinant zoster vaccine (RZV) offers effective protection against herpes zoster, with evidence supporting its efficacy in older adults aged ≥50 years, and adults aged ≥18 years at increased risk of herpes zoster. In the pivotal phase 3 ZOE trials, RZV demonstrated >90% efficacy against herpes zoster, PHN, and non-PHN complications in adults aged ≥50 years. Additionally, it provided durable protection with >80% efficacy for up to 10 years following the initial vaccination [58–60]. RZV efficacy and safety were also found to be comparable in populations with selected medical conditions at enrolment, including respiratory disorders such as COPD [61]. The results of this study add to the evidence on the burden of herpes zoster in adults with COPD, and support the GOLD recommendations for herpes zoster vaccination (currently GOLD level of evidence B) of all adults with COPD aged ≥50 years.

There were several limitations to this systematic literature review. Study data were from electronic databases using ICD/equivalent coding, without data on disease severity (some used ICS prescriptions to identify more severe COPD), lifestyle factors or other comorbidities, thus limiting adjustment for confounders. Herpes zoster incidence may be underestimated, as electronic databases capture people seeking healthcare and exclude uninsured people potentially in worse health, while general practitioner databases generally exclude institutionalised people. However, a study from the USA in older adults found a low proportion (<5%) not seeking healthcare for herpes zoster [62]. Some studies defined cases with ICD/equivalent codes plus treatment prescriptions, which could have included more severe cases while excluding less severe cases not on medication. Comparisons between studies were difficult due to different methods used, and pooled estimates had high heterogeneity and wide prediction intervals, due to the small number of studies included, warranting cautious interpretation. Studies had varied settings (single regions to nationwide studies), sample sizes (735 to >7 million overall, and 41 to >309 000 with COPD), and study periods (from 1997 to 2021), affecting generalisability and comparability of results, and strength of the pooled estimates.

Gaps in the literature were identified. No studies assessed the impact of COPD severity on herpes zoster development, and only a few studies reported on COPD treatments’ impact on herpes zoster development. Limited data were available on herpes zoster complications other than PHN in COPD, and on mortality outcomes. The data identified on COPD exacerbations following herpes zoster and on HCRU and costs used different outcome measures, making comparisons difficult. Finally, all studies were conducted in high-income countries, mainly in Europe.

### Conclusion

This is the first systematic literature review and meta-analysis focusing on herpes zoster burden in adults with COPD. Adults with COPD had a higher risk of developing herpes zoster and its complications (*e.g.* PHN). Herpes zoster added to the health and economic burden of COPD and was associated with significant increases in HCRU and costs. Herpes zoster vaccines are available for adults, including for those with comorbidities such as COPD, and should be offered as part of integrated respiratory care in accordance with national and international (*e.g.* GOLD [1], Advisory Committee on Immunization Practices [63], and the Standing Committee on Vaccination (STIKO) [64]) recommendations.

**Points for clinical practice**

- A global systematic review and meta-analysis assessed herpes zoster burden and risks in adults with COPD.
- Pooled herpes zoster incidence rate per 1000 person-years in adults with COPD aged ≥18 years was 10.98 (95% CI 8.28–14.56), increasing to 13.95 (95% CI 10.80–18.02) in adults aged ≥50 years.
- COPD was a risk factor for developing herpes zoster (pooled risk ratio 1.49, 95% CI 1.17–1.89); as was COPD treated with corticosteroids (1.86, 95% CI 1.28–2.69).
- Adults with COPD had an increased risk of herpes zoster complications such as postherpetic neuralgia (pooled rate ratio 1.50, 95% CI 1.10–2.04).
- Herpes zoster resulted in significantly higher healthcare resource use and costs.

## Supplementary material

**Please note:** supplementary material is not edited by the Editorial Office, and is uploaded as it has been supplied by the author. Supplementary material ERR-0167-2025.SUPPLEMENT Plain language summary ERR-0167-2025.SUPPLEMENT
