# Supplementary material for: Global herpes zoster burden in adults with COPD: a systematic review and meta-analysis
Source: Eur Respir Rev. 2026 Feb 4;35(179):250167. doi: 10.1183/16000617.0167-2025 (PMC12883191; doi:10.1183/16000617.0167-2025)
Supplement: Supplementary file 1 [file ERR-0167-2025.SUPPLEMENT.pdf]

Supplemental files

Manuscript: Global herpes zoster burden in adults with chronic obstructive pulmonary disease: a systematic review and meta-analysis

Alvaro A. Cruz,[a] Kevin J. Mortimer,[b,c,d] Ingrid T. Sepúlveda-Pachón,[e] Hilde Vroling,[f] Charles Williams[g]

[a] ProAR Foundation and Federal University of Bahia, Salvador, Brazil

[b] Cambridge Africa, Department of Pathology, University of Cambridge, UK

[c] Department of Paediatrics and Child Health, School of Clinical Medicine, College of Health Sciences, University of KwaZulu Natal, Durban, South Africa

[d] Respiratory Medicine, Aintree University Hospital, Liverpool, UK

[e] P95 Epidemiology & Pharmacovigilance, Bogotá, Colombia

[f] Pallas health research & consultancy, a P95 company, Rotterdam, Netherlands

[g] GSK, Wavre, Belgium

File S1. Search strategies

File S2. Quality control and assessment

File S3. Excluded articles by reason for exclusion

Table S1. Characteristics of COPD studies included

Table S2. Reported HZ incidence in adults with COPD

HZ incidence - Additional meta-analysis results

Figure S1. HZ incidence rate in COPD by age groups

Table S3. Reported risk of HZ in adults with COPD

HZ risk - Additional meta-analysis results and funnel plots

Figure S2 a1) Pre-specific sensitivity analysis: COPD as risk factor for HZ in adults ≥18 years (broader inclusion of additional studies [1-3])

a2) Funnel plot and Egger's test for studies reporting COPD as risk factor for HZ in adults ≥18 years (broader criteria)

b) Meta-analysis results of COPD as a risk factor for HZ in adults aged ≥50 years

c) Subgroup analysis: COPD as risk factor for HZ in ages 50–59, 60–69 and ≥70 years

Table S4. Healthcare resource use and costs

File S1. Search strategies

MEDLINE (in PUBMED)

#1: Search string for HZ
"Herpes Zoster"[Mesh] OR zoster[tw] OR shingle*[tw]
#2: Search string for asthma/COPD
"Pulmonary Disease, Chronic Obstructive"[Mesh] OR COPD[tiab] OR “chronic obstructive pulmonary disease”[tiab] OR COAD[tiab] OR “chronic obstructive airway disease”[tiab] OR “chronic obstructive lung disease”[tiab] OR “chronic bronchitis”[tiab] OR emphysema[tiab] OR “chronic airflow obstruction”[tiab] OR "Asthma"[Mesh] OR asthma*[tiab] OR “bronchial asthma”[tiab] OR wheez*[tiab]
#1 AND #2
Limits: from January 1st, 2003 until the search date of February 17th, 2024

EMBASE

#1 Search string for HZ
‘Herpes Zoster’/exp OR ‘herpes zoster’:ti:ab OR zoster:ti:ab OR shingle*:ti:ab
#2 Search string for asthma/COPD
'chronic obstructive lung disease'/exp OR COPD:ti:ab OR ‘chronic obstructive pulmonary disease’:ti:ab OR COAD:ti:ab OR ‘chronic obstructive airway disease’:ti:ab OR ‘chronic obstructive lung disease’:ti:ab OR ‘chronic bronchitis’:ti:ab OR emphysema:ti:ab OR ‘chronic airflow obstruction’:ti:ab OR 'asthma'/exp OR asthma*:ti:ab OR ‘bronchial asthma’:ti:ab OR wheez*:ti:ab
#1 AND #2
Limits: publication type: article, article in press or review; from January 1st, 2003 until the search date of February 17th, 2024

File S2. Quality control and assessment

The following quality control measures were used. All titles and abstracts were screened by two independent researchers. The results were compared and discussed, and differences between the two researchers concerned <5% of the articles. Regarding full-text articles, 30% were checked for relevancy in duplicate by two researchers independently. The results were compared and discussed early in the process, and in case of discrepancy, a third reviewer was consulted.

Joanna Briggs Institute: critical appraisal checklist for cohort studies

	Yes	No	Unclear	Not applicable
1. Were the two groups similar and recruited from the same population?	□	□	□	□
2. Were the exposures measured similarly to assign people to both exposed and unexposed groups?	□	□	□	□
3. Was the exposure measured in a valid and reliable way?	□	□	□	□
4. Were confounding factors identified?	□	□	□	□
5. Were strategies to deal with confounding factors stated?	□	□	□	□
6. Were the groups/participants free of the outcome at the start of the study (or at the moment of exposure)?	□	□	□	□
7. Were the outcomes measured in a valid and reliable way?	□	□	□	□
8. Was the follow up time reported and sufficient to be long enough for outcomes to occur?	□	□	□	□
9. Was follow up complete, and if not, were the reasons to loss to follow up described and explored?	□	□	□	□
10. Were strategies to address incomplete follow up utilized?	□	□	□	□
11. Was appropriate statistical analysis used?	□	□	□	□

Quality assessment results: cohort studies

**Item**	**Batram 2021 [4]**	**Calabria 2020 [5]**	**Ghaswalla 2021 [6]**	**Imafuku 2019 [7]**	**Imafuku 2020 [1]**	**Langan 2013 [3]**	**Muñoz-Quiles, 2018[8]**	**Thompson-Leduc 2022 [9]**	**Yang 2011 [10]**
1	Yes	No	No	NA	NA	No	No	No	Yes
2	Yes	Yes	Yes	NA	NA	Yes	Yes	Yes	Yes
3*	Yes	No	No	Yes	Yes	Yes	Yes	No	No
4	Yes	No	Yes	No	Yes	Yes	Yes	Yes	Yes
5	Yes	No	Yes	No	Yes	Yes	Yes	Yes	Yes
6	Yes	Yes	Yes	Yes	Yes	Yes	Yes	Yes	Yes
7	No	No	Yes	No	No	Yes	Yes	Yes	No
8	Yes	Yes	Yes	Yes	Yes	Yes	Yes	Yes	Yes
9	No	No	No	No	No	No	No	No	No
10	Yes	No	No	Yes	Yes	Yes	No	Yes	Yes
11	No	Yes	Yes	Yes	Yes	Yes	Yes	Yes	Yes

*Yes: COPD defined using ICD/equivalent coding without restriction to certain drug prescriptions; COPD: chronic obstructive pulmonary disease; ICD: International Classification of Diseases

Joanna Briggs Institute: critical appraisal checklist for case–control studies

	Yes	No	Unclear	Not applicable
1. Were the groups comparable other than the presence of disease in cases or the absence of disease in controls?	□	□	□	□

2. Were cases and controls matched appropriately?	□	□	□	□
3. Were the same criteria used for identification of cases and controls?	□	□	□	□
4. Was exposure measured in a standard, valid and reliable way?	□	□	□	□
5. Was exposure measured in the same way for cases and controls?	□	□	□	□
6. Were confounding factors identified?	□	□	□	□
7. Were strategies to deal with confounding factors stated?	□	□	□	□
8. Were outcomes assessed in a standard, valid and reliable way for cases and controls?	□	□	□	□
9. Was the exposure period of interest long enough to be meaningful?	□	□	□	□
10. Was appropriate statistical analysis used?	□	□	□	□

Quality assessment results: case–control studies

Item	Forbes 2014 [11]	Joesoef 2012 [12]	Kwon 2016 [13]	Lasserre 2012 [14]	Morena 2023 [2]	Schmidt 2017 [15]
1	Yes	Yes	Yes	Yes	Yes	Yes
2	Yes	Yes	Yes	Yes	No	Yes
3	Yes	Yes	Yes	Yes	No	Yes
4*	Yes	Yes	No	No	No	Yes
5	Yes	Yes	Yes	Yes	No	Yes
6	Yes	Yes	Yes	Yes	Yes	Yes
7	Yes	Yes	Yes	Yes	Yes	Yes
8	Yes	Yes	Yes	Yes	No	No
9	Yes	No	Yes	No	No	Yes
10	Yes	Yes	Yes	No	No	Yes

*Yes: COPD defined using ICD/equivalent coding without restriction to certain drug prescriptions; COPD: chronic obstructive pulmonary disease; ICD: International Classification of Diseases

Joanna Briggs Institute: critical appraisal checklist for cross–sectional studies

	Yes	No	Unclear	Not applicable
1. Were the criteria for inclusion in the sample clearly defined?	□	□	□	□
2. Were the study subjects and the setting described in detail?	□	□	□	□
3. Was the exposure measured in a valid and reliable way?	□	□	□	□
4. Were objective, standard criteria used for measurement of the condition?	□	□	□	□
5. Were confounding factors identified?	□	□	□	□
6. Were strategies to deal with confounding factors stated?	□	□	□	□
7. Were the outcomes measured in a valid and reliable way?	□	□	□	□
8. Was appropriate statistical analysis used?	□	□	□	□

Quality assessment results: cross–sectional studies

Item	Queenan 2018 [16]	Yawn 2022 [17
1	No	No
2	No	Yes
3	Yes	No
4*	Yes	No
5	No	NA
6	Yes	NA
7	Yes	No

8	Yes	Yes

*Yes: COPD defined using ICD/equivalent coding without restriction to certain drug prescriptions; COPD: chronic obstructive pulmonary disease; ICD: International Classification of Diseases

Joanna Briggs Institute: critical appraisal checklist for prevalence/incidence studies

	Yes	No	Unclear	Not applicable
1. Was the sample frame appropriate to address the target population?	□	□	□	□
2. Were study participants sampled in an appropriate way?	□	□	□	□
3. Was the sample size adequate?	□	□	□	□
4. Were the study subjects and the setting described in detail?	□	□	□	□
5. Was the data analysis conducted with sufficient coverage of the identified sample?	□	□	□	□
6. Were valid methods used for the identification of the condition?	□	□	□	□
7. Was the condition measured in a standard, reliable way for all participants?	□	□	□	□
8. Was there appropriate statistical analysis?	□	□	□	□
9. Was the response rate adequate, and if not, was the low response rate managed appropriately?	□	□	□	□

Quality assessment results: prevalence/incidence studies

**Item**	**Esteban-Vasallo 2014 [18]**	**Forbes 2016 [19**	**Gil-Prieto 2011 [20]**	**Muñoz-Quiles 2018 [21]**	**Sodergren 2024 [22]**
1	Yes	Yes	Yes	Yes	No
2	Yes	Yes	Yes	Yes	No
3	Yes	Yes	Yes	Yes	Yes
4	Yes	Yes	No	Yes	No
5	Yes	Yes	Yes	Yes	No
6	Yes	Yes	Yes	Yes	No
7	Yes	Yes	Yes	Yes	Yes
8	Yes	Yes	Yes	Yes	Yes
9	Yes	No	NA	No	NA

*Yes: COPD defined using ICD/equivalent coding without restriction to certain drug prescriptions; COPD: chronic obstructive pulmonary disease; ICD: International Classification of Diseases

File S3. Excluded articles by reason for exclusion

Note: Studies in both COPD and asthma populations were included, however, only data in adults with COPD were assessed for this study. Any outcomes that were not reported separately for COPD and asthma were excluded. Results show that none of the studies identified provided outcomes in a combined asthma/COPD population.

Not outcome of interest included (n=4):

1. Ernst P, Dell'Aniello S, Mikaeloff Y, Suissa S. Risk of herpes zoster in patients prescribed inhaled corticosteroids: a cohort study. BMC Pulm Med. 2011;11:59.
2. Iglar K, Kopp A, Glazier RH. Herpes zoster as a marker of underlying malignancy. Open Med. 2013;7(2):e68-73.
3. Malkud S, Dyavannanavar V, Purnachandra, Murthy KS. Clinical and morphological characteristics of herpes zoster - A study from tertiary care centre. J Pak Assoc Dermatol. 2017;26(3):219-22.
4. Omatsu Y, Shimizu Y, Haruki T, Inoue Y, Miyazaki D. Effect of atopic conditions on development and recurrences of infectious keratitis. Allergol Int. 2024 Feb 2:S1323-8930(24)00009-1.

Wrong population (n=10):

1. Wang XX, Zhang Y, Fan BF. Predicting postherpetic neuralgia in patients with herpes zoster by machine learning: a retrospective study. Pain Ther. 2020;9(2):627-35.
2. Risco Risco C, Herrador Z, Lopez-Perea N, Martinez-Urbistondo D, Del Villar Carrero RS, Masa-Calles J. Epidemiology of Herpes Zoster in the pre-vaccination era: establishing the baseline for vaccination programme's impact in Spain. Euro Surveill. 2023;28(8):2200390.
3. Bloechliger M, Reinau D, Spoendlin J, Chang SC, Kuhlbusch K, Heaney LG, et al. Adverse events profile of oral corticosteroids among asthma patients in the UK: cohort study with a nested case–control analysis. Respir Res. 2018;19(1):75
4. Bollaerts K, Alexandridou M, Verstraeten T. Risk factors for modified vaccine effectiveness of the live attenuated zoster vaccine among the elderly in England. Vaccine X. 2019;1:100007.
5. Borkar DS, Gonzales JA, Tham VM, Esterberg E, Vinoya AC, Parker JV, et al. Association between atopy and herpetic eye disease: results from the pacific ocular inflammation study. JAMA Ophthalmol. 2014;132(3):326-31.
6. Kim SY, Oh DJ, Choi HG. Asthma increases the risk of herpes zoster: a nested case–control study using a national sample cohort. Allergy Asthma Clin Immunol. 2020;16:52.
7. Lemmetyinen R, Karjalainen J, But A, Renkonen R, Pekkanen J, Haukka J, et al. Diseases with oral manifestations among adult asthmatics in Finland: a population-based matched cohort study. BMJ Open. 2021;11(12):e053133.
8. Liu B, Heywood AE, Reekie J, Banks E, Kaldor JM, McIntyre P, et al. Risk factors for herpes zoster in a large cohort of unvaccinated older adults: a prospective cohort study. Epidemiol Infect. 2015;143(13):2871-81.
9. Ogunjimi B, Buntinx F, Bartholomeeusen S, Terpstra I, De Haes I, Willem L, et al. Herpes zoster is associated with herpes simplex and other infections in under 60 year-olds. J Infect. 2015;70(2):171-7.
10. Peng YH, Fang HY, Wu BR, Kao CH, Chen HJ, Hsia TC, et al. Adult asthma is associated with an increased risk of herpes zoster: A population-based cohort study. J Asthma. 2017;54(3):250-7.

Wrong publication type (n=1):

1. Muñoz-Quiles C, López-Lacort M, Díez-Domingo J. Herpes zoster-associated resources consumption in chronic obstructive pulmonary disease patients. Value in Health. 2016;19(7):A603-A4.

SLR/meta-analysis (n=7):

1. Sarnes E, Crofford L, Watson M, Dennis G, Kan H, Bass D. Incidence and US costs of corticosteroid-associated adverse events: a systematic literature review. Clin Ther. 2011;33(10):1413-32.
2. Kawai K, Yawn BP. Risk factors for herpes zoster: a systematic review and meta-analysis. Mayo Clin Proc. 2017;92(12):1806-21.
3. Mareque M, Oyaguez I, Morano R, Casado MA. Systematic review of the evidence on the epidemiology of herpes zoster: incidence in the general population and specific subpopulations in Spain. Public Health. 2019;167:136-46.
4. Marra F, Parhar K, Huang B, Vadlamudi N. risk factors for herpes zoster infection: a meta-analysis. Open Forum Infect Dis. 2020;7(1):ofaa005.
5. Wu KK, Lee MP, Lee EB, Wu JJ. Risk of herpes zoster with IL-17 inhibitor therapy for psoriasis and other inflammatory conditions. J Dermatolog Treat. 2020;31(4):359-65.
6. Kwon JH, Wi CI, Seol HY, Park M, King K, Ryu E, et al. Risk, mechanisms and implications of asthma-associated infectious and inflammatory multimorbidities (AIMs) among individuals with asthma: a systematic review and a case study. Allergy Asthma Immunol Res. 2021;13(5):697-718.
7. Steinmann M, Lampe D, Grosser J, Schmidt J, Hohoff ML, Fischer A, Greiner W. Risk factors for herpes zoster infections: a systematic review and meta-analysis unveiling common trends and heterogeneity patterns. Infection. 2024 Jan 18.

Table S1. Characteristics of COPD studies included

Author, year Country	Study design Period	Study population	Setting	Total sample	COPD sample	COPD definition, severity/treatment	Comorbidities COPD	HZ definition	In/out-patients	Outcome measure
Batram, 2021 [4] Germany	Retrospective matched cohort 01/01/2008–31/12/2018	Patients aged ≥18 years, 37% males	Claims data from SHI, BARMER database	Year 2018: 7,008,996	Year 2018: 309,192	ICD-10 codes J44, J96<br><br>25.9% SCS usage (annual average)	NR	ICD/other codes (ICD-10-GM B02) + antiviral use (ATC: D06BB03, J05AB11, J05AB09, J05AB15, J05AB01, S01AD07, S01AD03)	Both	Incidence, risk factors for HZ (aOR)
Calabria, 2020 [5] Italy	Retrospective unmatched cohort 01/01/2013–31/12/2015	Patients aged ≥50 years with at least one risk factor for HZ (i.e. CVD, COPD, diabetes, immunosuppression), gender data NR	Fondazione ReS database	1,004,705	228,435	Pharmaceutical prescription (≥2 packs for ≥3 months code R03 [ex R03DC and R03BC]) AND/OR 1) ≥1 hospital admission with a main diagnosis of COPD (ICD-9: 490, 491.x, 492.x, 494.x, 496) OR 2) ≥1 hospital admission with a main diagnosis for COPD-related causes (518.81- 518.84, 786.0x, 786.2, 786.4) with secondary diagnosis of COPD. Excluding asthma 007, 493<br><br>NR	NR	ICD/other codes (ICD-9-CM 053.x; hospital admission only) +/- antiviral use (ATC code: J05AB15)	Both	Incidence, HCRU, LOS, costs

Esteban-Vasallo, 2014 [18] Spain	Retrospective passive surveillance 01/10/2009–31/12/2012	Patients aged ≥18 years with access to the Madrid Regional Public Health System, 47 % males	ECRPC in the IHC of the Madrid Regional Public Health System	5,244,402	88,003	ICPC codes R95, R79, R93 (ICPC-1)<br><br>NR	NR	ICD/other codes (ICPC S70 code + descriptive clinical text)	Outpatients	Incidence, risk factors for HZ (aIRR)
Forbes, 2014 [11] UK	Matched case–control 01/01/2000–31/12/2011	Patients aged ≥18 years, 40.6% males in HZ cases, and 39% males in controls	CPRD and HES database	694,295	27,016	Read codes (NR) for COPD, including chronic bronchitis and emphysema, prior to the index date and aged ≥35 years at first COPD diagnosis<br><br>NR	NR	ICD/other codes (Read codes NR; ICD-10: B02, B02.0, B02.1, B02.31, B02.7, B02.8, B02.9, G53.0)	Both	Incidence, risk factors for HZ (aOR)
Forbes, 2016 [19] UK	Retrospective passive surveillance 01/01/2000–31/12/2011	Patients ≥18 years old with first ever zoster, 41.8 % males	CPRD and HES database	119,413	5,060	Read codes (NR) for COPD, including chronic bronchitis and emphysema, prior to the index date and aged ≥35 years at first COPD diagnosis<br><br>NR	NR	ICD/other codes (Read code or ICD-10 NR)	Both	PHN prevalence, risk factors for PHN (aOR)
Ghaswalla, 2021 [6] US	Retrospective unmatched cohort 01/01/2013–31/12/2018	Patients ≥50 years old with ≥1 COPD diagnosis, 44.7% males	Optum CDM database	38,775	38,775	≥1 inpatient primary COPD claim OR ≥2 outpatient COPD claims (with one primary diagnosis) OR ≥1 outpatient COPD claim + COPD maintenance treatment (LAMA, LABA, ICS/LABA, LABA/LAMA, ICS/LABA/LAMA); COPD	24.2% asthma, 27.3% comorbidities potentially associated with HZ (RA, IBD, SLE, MS, psoriasis, CKD, Wegener’s granulomatosis, polymyalgia	ICD/other codes (ICD-9 053 excluding 053.1; ICD-10 B02 excluding B02.2)	Both	HCRU, costs, COPD ex-acerbation, vascular complica-tions (aIRR)

						codes ICD-9 491, 492, 496, ICD-10 J41, J42, J43, J44<br><br>ICS 51.18%, OCS 30.3%	rheumatica, GCA, ankylosing spondylitis, IPF, sarcoidosis, scleroderma, psoriatic arthritis), 4.7% immunosup-pression status*			
Gil-Prieto, 2011 [20] Spain	Retrospective passive surveillance 01/01/1998–31/12/2004	Patients ≥50 years with HZ, gender data NR	CMBD database	16,022	3,633	ICD-9 codes for COPD (codes NR)<br><br>NR	NR	ICD/other codes (ICD-9-CM 053; 053.0-053.9)	Inpatients	Hospital LOS, mortality, costs
Imafuku, 2019 [7] Japan	Retrospective unmatched cohort 01/2005–31/12/2014	Patients aged 18–74 years registered in the JMDC-CDB, 54.76% males	JMDC-CDB database	2,778,476	9,374	ICD-10 codes J41, J42, J43, J44<br><br>NR	NR	ICD/other codes (ICD-10 B02, B02.0, B02.1, B02.2, B02.3, B02.7, B02.8, and B02.9) + antiviral use (JMCD drug codes)	Both	Incidence
Imafuku, 2020** [1] Japan	Retrospective unmatched cohort 01/2005–31/12/2014	Patients aged 18–74 years registered in the JMDC-CDB, 54.76% males	JMDC-CDB database	2,778,476	9,374	ICD-10 codes J41, J42, J43, J44<br><br>NR	NR	ICD/other codes (ICD-10 B02, B02.0, B02.1, B02.2, B02.3, B02.7, B02.8, and B02.9) + antiviral use (JMCD drug codes)	Both	Incidence, risk factors for HZ (aHR)
Joesoef, 2012 [12] US	Matched case–control 01-01-2007/31-12-2007	Adults 20–64 years old, 45.8% males	MarketScan databases	675,350	4,501	ICD-9-CM codes 491.xx, 492.8<br><br>NR	NR	ICD/other codes (ICD-9-CM 053.xx)	Outpatients	Risk factors for HZ (aOR)

Kwon, 2016 [13] US	Matched case–control 01/01/2010-31/10/2011	Patients ≥50 years of age, 34% males	Rochester Epidemiology Project database	1,113	41	NR<br><br>NR	NR	ICD/other codes (ICD-9 053.xx) + medical record review	Both	Risk factors for HZ (OR)
Langan, 2013 [3] US	Retrospective unmatched cohort 01/01/2007–31/12/2009	Individuals ≥65 years old in a 5% random sample of Medicare, 32.4% males	Medicare database	766,330	212,6 35	ICD-9-CM codes on different days in the records (codes NR)<br><br>NR	NR	ICD/other codes (ICD-9-CM codes for HZ, excluding codes for PHN) + antiviral use (acyclovir, famciclovir or valacyclovir) (codes NR)	Both	Incidence, risk factors for HZ (aHR)
Lasserre, 2012 [14] France	Matched case–control 04/2009–09/2010	Patients ≥50 years of age, 41% males	GPs belonging to the French 'Sentinel's network' (FSN)	750	41	GP questionnaire, not further specified<br><br>NR	NR	Clinical diagnosis by GP (acute and painful vesicular rash with dermatomal distribution)	Outpatien ts	Risk factors for HZ (OR)
Morena, 2023 [2] Spain	Unmatched case–control 01/2012–12/2020	Adults >20 years, gender NR	Electronic health records in Castilla-La-Mancha Public Health System (SESCAM)	2,602,60 8	74,50 2	NR<br><br>COPD with HZ: 86.6% received ICS as a baseline treatment, 88.5% treated with systemic corticosteroids very irregularly (mostly sporadically)	COPD with HZ: arterial hypertension 77.2%, DM 41.7%, dyslipidaemia 60.2%, depression 21.5%	NR	Both	Risk factors for HZ and PHN (aOR), PHN prevalence
Muñoz-Quiles, 2018 [8] Spain	Retrospective unmatched cohort 01/01/2009–31/12/2014	Individuals ≥50 years old insured by the RHS and living in Valencia, 46% males	Primary care electronic medical records (SIA) and the minimum basic dataset (MBDS; hospital DB) of the	2,289,48 5	161,3 17	ICD9-CM code for COPD (491, 492 or 496)<br><br>ICS: 18.4%	20.5% heart failure, 32.8% DM	ICD/other codes (ICD-9-CM 053.x)	Both	Incidence, recurrence, risk factors for HZ (aRR), HCRU, COPD ex-acerbation

			Regional Health System							
Muñoz-Quiles, 2018 [21] Spain	Retrospective passive surveillance 01/01/2009–31/12/2014	Individuals ≥50 years old insured by the RHS and living in Valencia, 46% males	Primary care electronic medical records (SIA) and the minimum basic dataset (MBDS; hospital DB) of the Regional Health System	2,289,485	161,317	ICD9-CM code for COPD (491, 492 or 496)<br><br>NR	NR	ICD/other codes (ICD-9-CM 053.x)	Both	PHN prevalence, risk factors for PHN (aRR)
Queenan, 2018 [16] Canada	Cross–sectional 01/01/2015–31/12/2015	Patients aged ≥18 years, 43% males	CPCSSN sentinel surveillance; data from networks in Alberta, Manitoba, Ontario and Newfoundland	1,018,641	24,607	Validated CPCSSN case definitions (obstructive chronic bronchitis, emphysema, chronic airway obstruction. Classification requires more than an X-ray or spirometry finding and must include either a specific or similar diagnosis of COPD. Excluded: all types of asthma)<br><br>NR	NR	ICD/other codes (ICD-9 053.xx)	Outpatients	Incidence, risk factors for HZ (aRR)

Schmidt, 2017 [15] Denmark	Nested matched case–control 01/01/1997–31/12/2013	All patients ≥40 years old in hospital-based inpatient, outpatient specialty clinic, and emergency room records, 39.02% males in HZ group; gender in controls NR	Danish National Prescription and Patient Registries	1,134,138	51,431	Incident diagnoses of chronic bronchitis and/or emphysema at ≥35 years of age (ICD-8 491, 492 or ICD-10 DJ41, DJ42, DJ43, DJ45)<br><br>NR	NR	ICD/other codes (ICD-8: 053, ICD-10: B02, G051I/M, H031F, H131M, H190D, H192D, H192J, H220C, H621B) + antiviral use (ATC: J05AB01, J05AB09, J05AB11)	Both	Risk factors for HZ (aOR)
Södergren, 2024 [22] Sweden	Retrospective passive surveillance 2005–2021	Individuals ≥18 years old residing in Västra Götaland region between 2005 and 2021, 41% male	VEGA database (primary care, secondary/specialised care and inpatient care) and Digitalis register (prescriptions)	Total: NR HZ: 75,538	NR	≥1 healthcare visit with a registered ICD-10 code ((J41), J42-J44; unclear why the first code is between brackets) prior to the HZ index event and after 2005<br><br>NR	NR	≥1 primary or secondary diagnosis (ICD-10 code B02) between 2005 and 2021, without a prior HZ diagnosis during the preceding 365 days	Both	Incidence, PHN prevalence
Thompson-Leduc, 2022 [9] US	Retrospective unmatched cohort 01/01/2013–31/12/2018	Patients ≥40 years old with no claims for HZ vaccines or HZ, PHN or HZO at the index date; 46.7% males	Optum CDM database	9,805,495	161,970	≥1 inpatient primary COPD claim OR ≥2 outpatient COPD claims within 12 months (with one primary diagnosis) OR ≥1 outpatient COPD claim + COPD maintenance treatment within 12 months (LAMA, LABA, ICS/LABA, LABA/LAMA, ICS/LABA/LAMA); COPD	16.2% asthma, 0.4% use of immunosuppressant, 72.1% CPD, 20.6% CHF, 29.4% DM, 16.6% PVD, 14.3% renal disease, 11.4% CVD, 9.8% any malignancy***, 7.3% MI, 3.9%	ICD/other codes (ICD-9-CM 053 excluding 053.1; ICD-10-CM B02 excluding B02.2)	Both	Incidence (including PHN), risk factors for HZ and PHN (aIRR)

						codes ICD-9 491, 492, 496, ICD-10 J41, J42, J43, J44<br><br>OCS: 35.4% (short-term: 89.9%/long-term: 10.1%), ICS: 30.0%	mild liver disease, 3.9% rheumatologic disease, 3.5% dementia, 1.8% metastatic solid tumour, 1.6% peptic ulcer disease, 1.2% hemiplegia or paraplegia, 0.5% moderate or severe liver disease, 0.2% AIDS/HIV			
Yang, 2011 [10] Taiwan	Retrospective matched cohort 01/01/2004–31/12/2006	Patients ≥50 years old; 69% males	The Longitudinal Health Insurance Database	42,430	8,486	≥2 outpatient visits for COPD within 12 months OR a hospital admission with a primary diagnosis of COPD (ICD-9-CM 491, 492, 496) + COPD treatment use >1 month (inhaled and oral β2-agonists, anticholinergic agents, corticosteroids and theophylline)<br><br>ICS only: 6%, OCS: 17.5%	17.8% DM, 2.4% rheumatic disease, 5.9% cancer	ICD/other codes (ICD-9 codes NR)	Both	Incidence, risk factors for HZ (aHR)
Yawn, 2022 [17] US	Cross–sectional 21/09/2020–10/11/2020	COPD PPRN registrants aged ≥50 years with self-reported COPD who had completed the	PPRN survey	735	735	Self-reported physician diagnosis of COPD<br><br>Mean (SD) COPD Assessment Test score: 19.6 (8.0)	NR	Self-reported using question 'Have you ever had shingles or herpes zoster?' Defined in the survey as 'a disease causing a	Outpatients	Proportion HZ, HZO and PHN prevalence, HCRU, COPD ex-acerbation

		baseline survey; 40.3% males						blistering skin rash that is often painful and usually in a band-like area on one side of the body’		

aHR: adjusted hazard ratio; aIRR: adjusted incidence rate ratio; AIDS/HIV: acquired immunodeficiency syndrome/human immunodeficiency virus; aOR: adjusted odds ratio; ATC: anatomical therapeutic chemical; CM: clinical modification; CHF: congestive heart failure; CKD: chronic kidney disease, CMBD: Conjunto Mínimo Básico de Datos; COPD: chronic obstructive pulmonary disease; CPCSSN: Canadian Primary Care Sentinel Surveillance Network; CPD: chronic pulmonary disease; CPRD: Clinical Practice Research Datalink; CVD: cardiovascular disease; DB: database; DM: diabetes mellitus; ECRPC: electronic clinical records in primary care; GCA: giant cell arteritis; GM: German modification; GP: general practitioner; HCRU: health care resources utilisation; HES: Hospital Episode Statistics; HIRA-NSC: Health Insurance Review And Assessment Service—National Sample Cohort; HZ: herpes zoster; HZO: herpes zoster ophthalmicus; IBD: inflammatory bowel disease (Crohn’s disease, ulcerative colitis); ICD: International Classification of Diseases; ICPC: International Classification for Primary Care; ICS: inhaled corticosteroids; IHC: individualised health card; IPF: idiopathic pulmonary fibrosis; JMDC-CDB: Japan Medical Data Center claims database; LABA: long-acting beta 2-agonist; LAMA: long-acting muscarinic antagonists; LOS: length of stay; MBDS: minimum basic data set; MI: myocardial infarction; MS: multiple sclerosis; NR: not reported; OCS: oral corticosteroids; Optum CDM: Optum’s de-identified Clinformatics Data Mart Database; PHN: postherpetic neuralgia; PPRN: Patient-Powered Research Network; PVD: peripheral vascular disease; RA: rheumatoid arthritis; ReS: Ricerca e Salute – Research and Health Foundation; SD: standard deviation; SESCAM: Servicio de Salud de Castilla-La Mancha; SHI: statutory health insurance; SIA: ambulatory information system; SLE: systemic lupus erythematosus; UK: United Kingdom; US: United States. *Immunosuppression status (solid organ transplantation (SOT), haematopoietic stem cell transplantation (HSCT), chemotherapy for solid and haematological malignancies in the previous 6 months, use of immunosuppressants, autoimmune conditions receiving biologics, symptomatic HIV disease (i.e. AIDS))

**Imafuku et al. 2020 and Imafuku et al. 2019 used the same study methodology and study population. Imafuku et al. 2020 reported on further analyses (stratified incidence data and risk factor results); ***Any malignancy, including leukaemia and lymphoma, but not malignant neoplasms of the skin.

Table S2. Reported HZ incidence in adults with COPD

Country<br>Study period	Age (years)	Follow-up time	Stratified by	Incidence	95% CI
**Incidence rate per 1 000 person-years**					
Esteban-Vasallo 2014 [18] Spain 2009–2012	≥18	NR	Both overall	11.38	NR
			Male	10.80	NR
			Female	12.87	NR
Batram 2021 [4] Germany 2008–2018	≥18	11 years	Year: 2008	8.18	NR
			Year: 2009	8.61	NR
			Year: 2010	8.14	NR
			Year: 2011	8.32	NR
			Year: 2012	7.43	NR
			Year: 2013	9.06	NR
			Year: 2014	8.29	NR
			Year: 2015	8.15	NR
			Year: 2016	8.41	NR
			Year: 2017	8.52	NR
			Year: 2018	8.71	NR
Forbes 2014 [11] UK 2000–2011	≥18	Mean 8.6 years	18–49 years	2.31	1.40–3.84
			50–59 years	5.62	2.44–12.94
			60–69 years	9.19	4.09–20.62
			≥70 years	11.54	5.08–26.20
Imafuku 2019 & 2020 [1, 7] Japan 2005–2014	18–74	Mean 4.88 years	18–74 years overall	7.25	6.06–8.6
			18–49 years	5.97	4.63–7.59
			50–59 years	9.71	7.03–13.08
			60–64 years	6.92	3.32–12.73
			65–74 years	11.66	5.82–20.87
			Male	7.07	5.61–8.80
			Female	7.55	5.62–9.92
Thompson-Leduc 2022 [9] US 2013–2018	≥40	36.6 months	≥40 overall	13.0	NR
			40–49	9.5	NR
			50–59	11.2	NR
			60–69	12.2	NR
			70–79	14.1	NR
			≥80	14.1	NR
Yang 2011 [10] Taiwan 2004–2006	≥50	27.7 months	≥50 overall	16.4	NR
			50–59	14.26	NR
			60–69	15.94	NR
			≥70	17.43	NR
			No CS	14.27	NR
			ICS	18.37	NR
			Oral CS	26.26	NR
Langan 2013 [3] US 2007–2009	≥65	359,878 person-years	HZ definition: ICD codes	17.7	17.2–18.1
			HZ definition: ICD codes + antiviral use	11.4	11.0–11.7
**Cumulative incidence per 1 000 population**					
Queenan 2018 [16]	≥18	NR	Both	6.9	NR

Canada 2015			Male	5.8	NR
			Female	7.9	NR
Södergren 2024 [22] Sweden 2014–2019*	≥18	NR	≥18 overall	12.3	11.7–12.9
			18–49	1.0	0.6–1.4
			50–64	6.2	5.4–6.9
			≥65	20.4	19.2–21.5
Calabria 2020 [5] Italy 2013–2015	≥50	2 years	NA, overall	3.4	NR
Muñoz-Quiles 2018 [8] Spain 2009–2014	≥50	6 years	≥50 overall, no ICS	11.1	10.7–11.4
			≥50 overall, ICS	13.0	12.3–13.8
			50–59, No ICS	7.4	6.7–8.1
			60–69, No ICS	10.3	9.7–10.9
			70–79, No ICS	12.0	11.4–12.6
			≥80, No ICS	12.8	12.1–13.5
			50–59, ICS	10.6	8.7–12.8
			60–69, ICS	11.6	10.2–13.1
			70–79, ICS	13.8	12.5–15.2
			≥80, ICS	14.2	12.8–15.7
			Male, no ICS	10.3	10.0–10.7
			Female, no ICS	12.6	12.0–13.2
			Male, ICS	11.7	10.8–12.7
			Female, ICS	14.6	13.5–15.9

95% CI: 95% confidence interval; COPD: chronic obstructive pulmonary disease; CS: corticosteroids; HZ: herpes zoster; ICD: International Classification of Diseases; ICS: inhaled corticosteroids; NA: not applicable; NR: not reported; OCS: oral corticosteroids; UK: United Kingdom; US: United States. *Entire study period is 2005–2021 but HZ incidence in COPD reported for 2014–2019.

HZ incidence - Additional meta-analysis results

Figure S1. HZ incidence rate in COPD by age groups

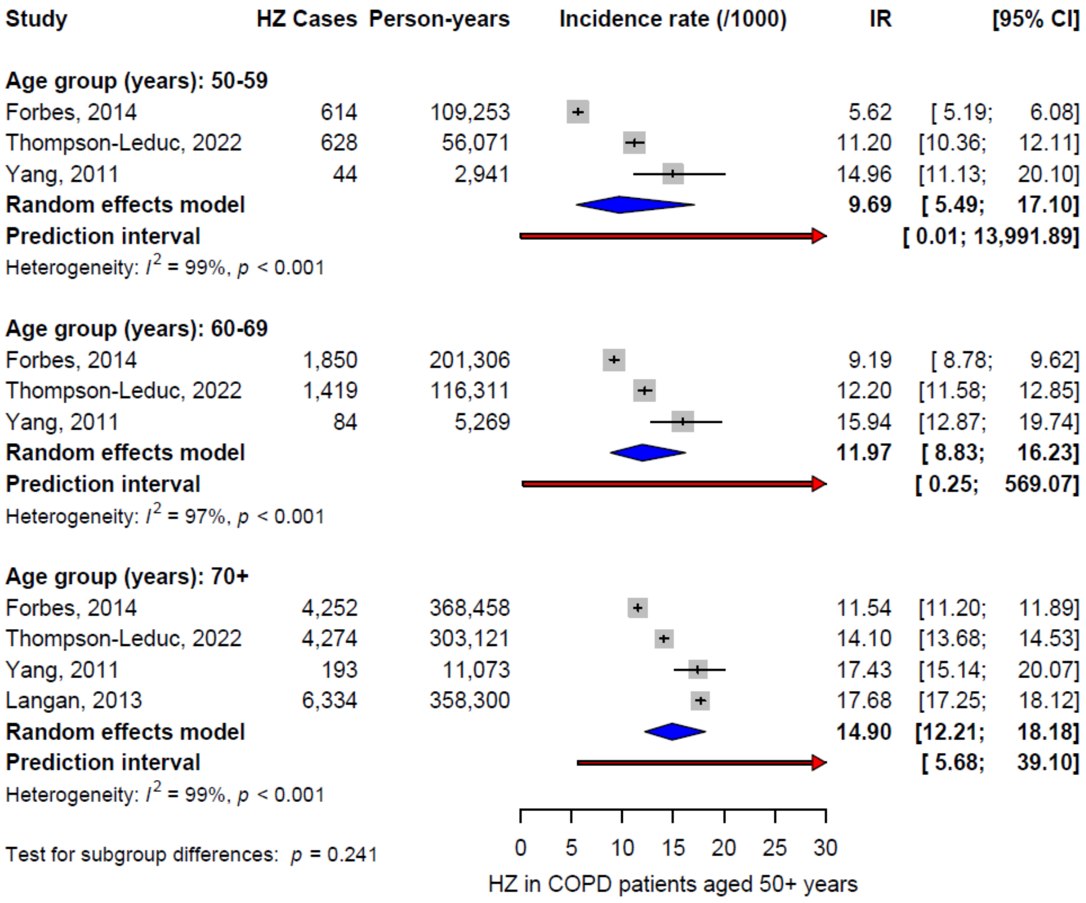

95% CI: 95% confidence interval; COPD: chronic obstructive pulmonary disease; HZ: herpes zoster; IR: incidence rate

Table S3. Reported risk of HZ in adults with COPD

Author, year<br>Country, period	Study design	Age group (years)	Stratified by	Value	95% CI	Matched/adjusted by
**HZ**						
Batram 2021 [4]<br>Germany<br>2008–2018 (2013 values presented)	Retrospective matched cohort	≥18	≥18 overall	aOR 1.10	1.05–1.14	Matched by age and sex; adjusted for each underlying comorbidity
			18–49<br>50–59<br>≥60	aOR 1.32<br>aOR 1.15<br>aOR 1.07	1.11–1.56<br>1.03–1.29<br>1.02–1.12	
			No systemic CS, 18+	aOR 1.03	0.98–1.08	
			No systemic CS, 18–49<br>No systemic CS, 50–59<br>No systemic CS, ≥60	aOR 1.23<br>aOR 1.09<br>aOR 1.00	1.00–1.50<br>0.95–1.26<br>0.94–1.06	
			Systemic CS, 18+	aOR 1.26	1.17–1.36	
			Systemic CS, 18–49<br>Systemic CS, 50–59<br>Systemic CS, ≥60	aOR 1.54<br>aOR 1.26<br>aOR 1.25	1.14–2.08<br>1.03–1.55<br>1.15–1.36	
Forbes 2014 [11]<br>UK 2000–2011	Matched case–control	≥18	≥18 overall	aOR 1.22	1.17–1.28	Matched by practice, sex, age, calendar time; adjusted for HIV, leukaemia, lymphoma, myeloma, HSCT, other unspecified cellular immune deficiencies, RA, SLE, IBD, asthma, CKD, depression, diabetes, smoking, alcohol, OCS, other immunosuppressive treatment and ICS
			18–49<br>50–59<br>60–69<br>≥70	aOR 1.11<br>aOR 1.29<br>aOR 1.37<br>aOR 1.30	0.80–1.54<br>0.65–2.53<br>0.71–2.66<br>0.68–2.51	Matched by practice, sex, age, calendar time; adjusted for HIV, leukaemia, lymphoma, myeloma, HSCT, other unspecified cellular immune deficiencies, RA, SLE, IBD, asthma, CKD, depression, diabetes, smoking and alcohol
Queenan 2018 [16]<br>Canada 2015	Cross–sectional	≥18	≥18 overall	aRR 1.83	1.28–2.62	Adjusted for age and sex
Morena, 2023 [2]<br>Spain 2012–2020	Unmatched case–control	>20	≥20 overall	aOR 1.16	1.13–1.19	Adjusted for sex, arterial hypertension, diabetes mellitus, dyslipidaemia and depression
Imafuku 2020 [1]<br>Japan 2005–2014	Retrospective unmatched cohort	18–74	18–74	aHR 1.24	1.04–1.47	Adjusted for age at index date, sex and for other IC or chronic conditions

Joesoef 2012 [12]<br>US 2007	Matched case–control	20–64	20–64	aOR 1.35	1.23–1.47	Matched by age and insurance plan; adjusted for sex, number of claims, and other serious chronic conditions
Schmidt 2017 [15]<br>Denmark 1997–2013	Matched nested case–control	≥40	Hospital + prescription-based HZ<br>Prescription-based HZ<br>Hospital-based HZ	aOR 1.20<br>aOR 1.16<br>aOR 2.05	1.16–1.24<br>1.12–1.20<br>1.83–2.29	Matched by sex and birth year; adjusted for comorbidities included RA, SLE, IBD, COPD, asthma, CKD, depression, diabetes, HIV, leukaemia, lymphoma, myeloma, HSCT, OID, ICS, oral CS, and other immunosuppressants
Thompson-Leduc 2022 [9]<br>US 2013–2018	Retrospective unmatched cohort	≥40	≥40 overall	aIRR 2.77	2.69–2.85	Adjusted for age at index, sex, region, insurance type, CCI score, asthma, use of chemotherapy, immunosuppressants and corticosteroids (short-term oral, long-term oral and inhaled)
			40–49<br>50–59<br>60–69<br>70–79<br>≥80	aIRR 2.50<br>aIRR 2.62<br>aIRR 2.71<br>aIRR 2.73<br>aIRR 2.63	2.11–2.96<br>2.42–2.85<br>2.56–2.86<br>2.60–2.87<br>2.46–2.80	
Kwon 2016 [13]<br>US 2010–2011	Matched case–control	≥50	≥50 overall	OR 1.48	0.77–2.84	Matched by age, gender and years since registration
Lasserre 2012 [14]<br>France 2009–2010	Matched case–control	≥50	≥50 overall	OR 0.63	0.29–1.30	Matched by age and sex
Muñoz-Quiles 2018 [8]<br>Spain 2009–2014	Retrospective unmatched cohort	≥50	50+ overall	aRR 1.45	1.41–1.50	Adjusted for sex, age, comorbidities (diabetes, heart failure), health department, calendar year and group variable (to avoid over-dispersion)
			ICS	aRR 1.61	1.52–1.71	
Yang 2011 [10]<br>Taiwan 2004–2006	Retrospective matched cohort	≥50	≥50 overall	aHR 1.68	1.45–1.95	Matched by age, sex and year of cohort entry; adjusted for age, sex, diabetes mellitus, rheumatic diseases, cancer, Charlson comorbidity index score, use of corticosteroids, monthly income, geographic region and urbanisation level
			50–59<br>60–69<br>≥70	aHR 1.85<br>aHR 1.65<br>aHR 1.68	1.27–2.70<br>1.24–2.20<br>1.38–2.04	
			No CS<br>ICS<br>OCS	aHR 1.67<br>aHR 2.09<br>aHR 3.00	1.43–1.96<br>1.38–3.16<br>2.40–3.75	
Langan 2013 [3]<br>US 2007–2009	Retrospective unmatched cohort	≥65	HZ by ICD codes	aHR 1.17	1.13–1.20	Adjusted for age, gender, race, immunosuppression, low income, IBD, kidney disease, diabetes mellitus, RA and SLE
			HZ by ICD codes + antiviral use	aHR 1.13	1.09–1.17	
Esteban-Vasallo 2014 [18]<br>Spain 2009–2012	Retrospective passive surveillance	≥18	Male	aIRR 1.28	1.22–1.34	Adjusted for age, origin, socioeconomic level, asthma, ischaemic heart disease, other cardiovascular diseases, diabetes, HIV-infection/AIDS, other immunodeficiency, and cancer
			Female	aIRR 1.23	1.15–1.31	

aHR: adjusted hazard ratio; AIDS: acquired immunodeficiency syndrome; aIRR: adjusted incidence rate ratio; (a)OR: (adjusted) odds ratio; aRR: adjusted risk ratio; CCI: Charlson Quan Comorbidity Index; CI: confidence interval; CKD: chronic kidney disease; COPD: chronic obstructive pulmonary disease; CS: corticosteroids; HIV: human immunodeficiency virus; HSCT: haematopoietic stem cell transplantation; HZ: herpes zoster; IBD: inflammatory bowel disease; ICD: International Classification of Diseases; ICS: inhaled corticosteroids; OCS: oral corticosteroids; OID: other immunosuppressive disease; PHN: postherpetic neuralgia; RA: rheumatoid arthritis; SHI: statutory health insurance; SLE: systemic lupus erythematosus; UK: United Kingdom; US: United States.

Note: Crude results were reported only when adjusted results were not available. ◊ Control was not non-COPD but 'no common chronic disease', defined as no diabetes, COPD, hypertension, depression, osteoarthritis, dementia, epilepsy, parkinsonism, any neoplasm or HIV/AIDS. °The primary definition of PHN included patients classified as having diagnosed, probable, or possible PHN, based on a validated algorithm of PHN within a US administrative database utilising diagnosis codes and prescription date. Diagnosed PHN: PHN codes (90–365 days post-zoster). Probable PHN: Zoster code and prescription consistent with PHN on same day (90–365 days post-zoster), nonspecific neuralgia code (90–365 days post-zoster), new anticonvulsant or capsaicin cream or lidocaine patch prescription (90–180 days post-zoster), new tricyclic antidepressants 90–180 days post-zoster with no other indication on the day of the prescription, plus evidence of the drug being prescribed for zoster or PHN previously. Possible PHN: New tricyclic antidepressants 90–180 days post-zoster with no other indication on the day of the prescription, new strong painkiller 90–180 days following zoster with no other indication on the day of the prescription, plus evidence of the drug being prescribed for zoster or PHN previously, nonspecific neuropathic pain code (90–365 days post-zoster).

Figure S2. HZ risk - Additional meta-analysis results and funnel plots

a1) Pre-specific sensitivity analysis: COPD as risk factor for HZ in adults ≥18 years (broader inclusion of additional studies [1-3])

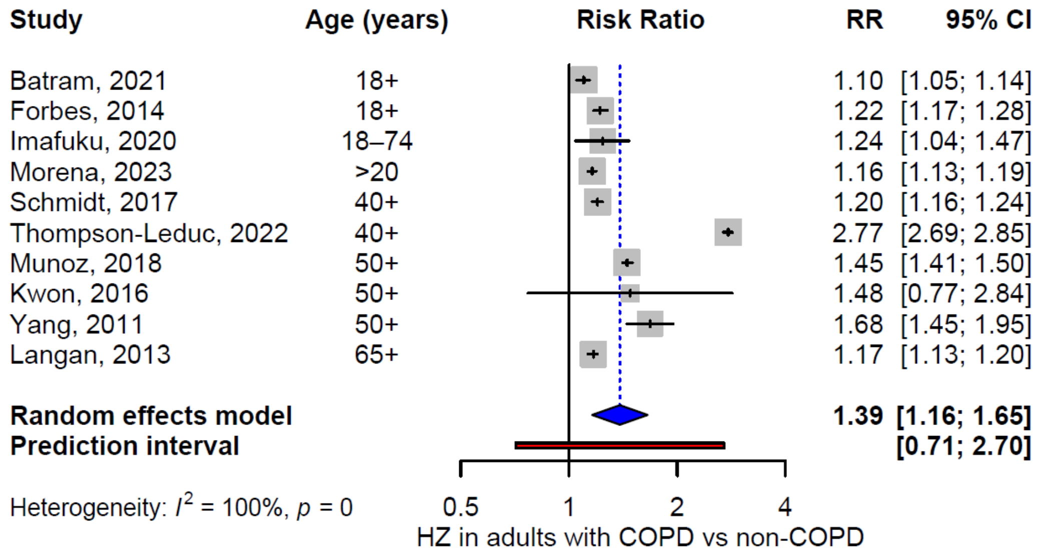

a2) Funnel plot and Egger's test for studies reporting COPD as risk factor for HZ in adults ≥18 years (broader criteria)

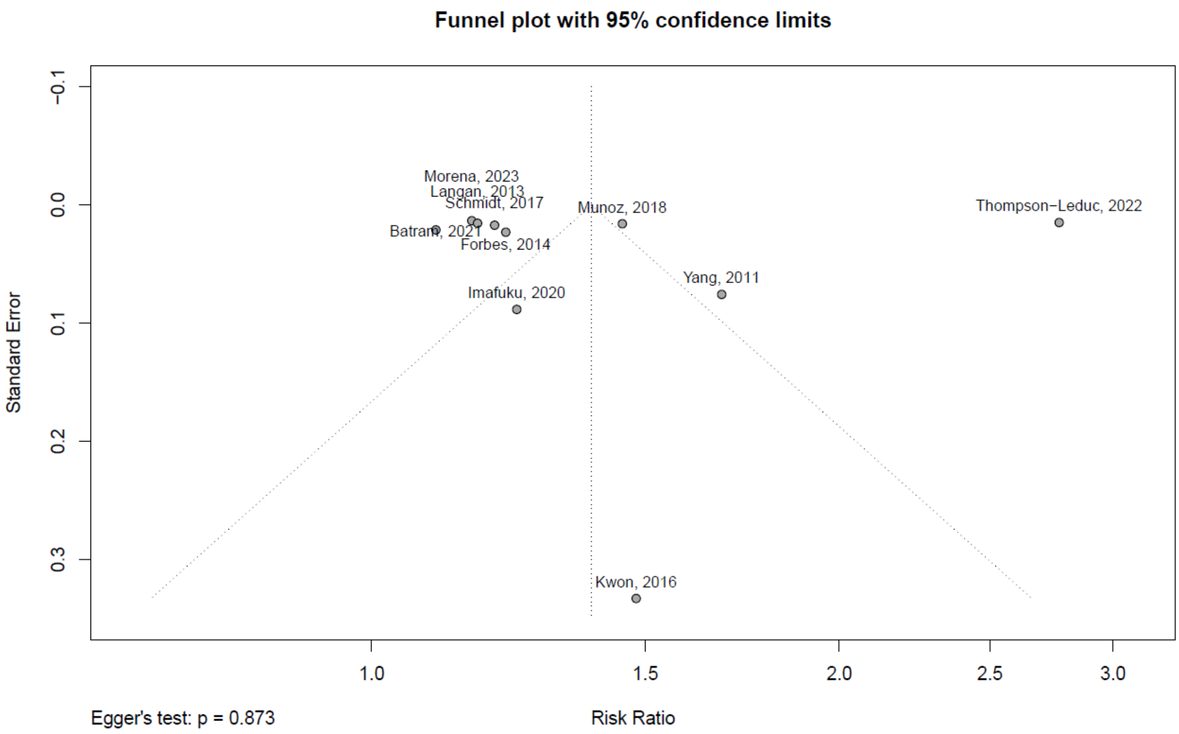

b) Meta-analysis results of COPD as a risk factor for HZ in adults aged ≥50 years

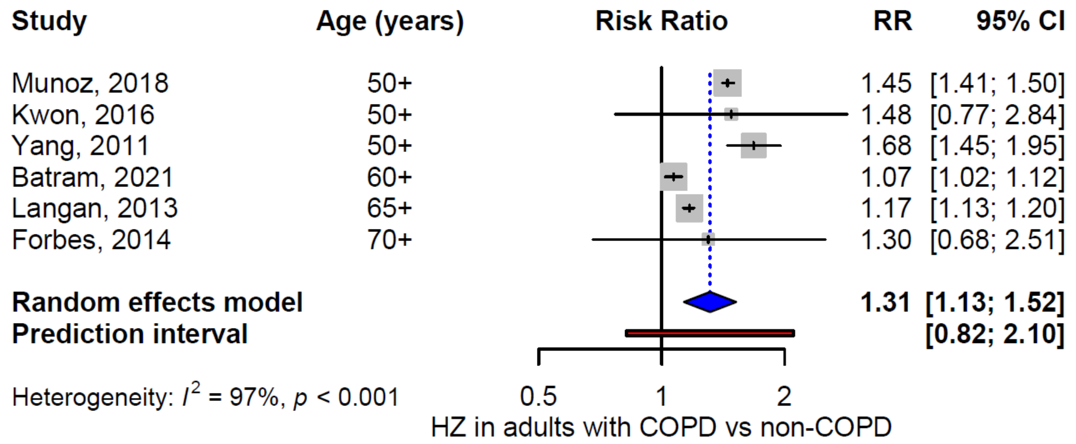

c) Subgroup analysis: COPD as risk factor for HZ in ages 50–59, 60–69 and ≥70 years

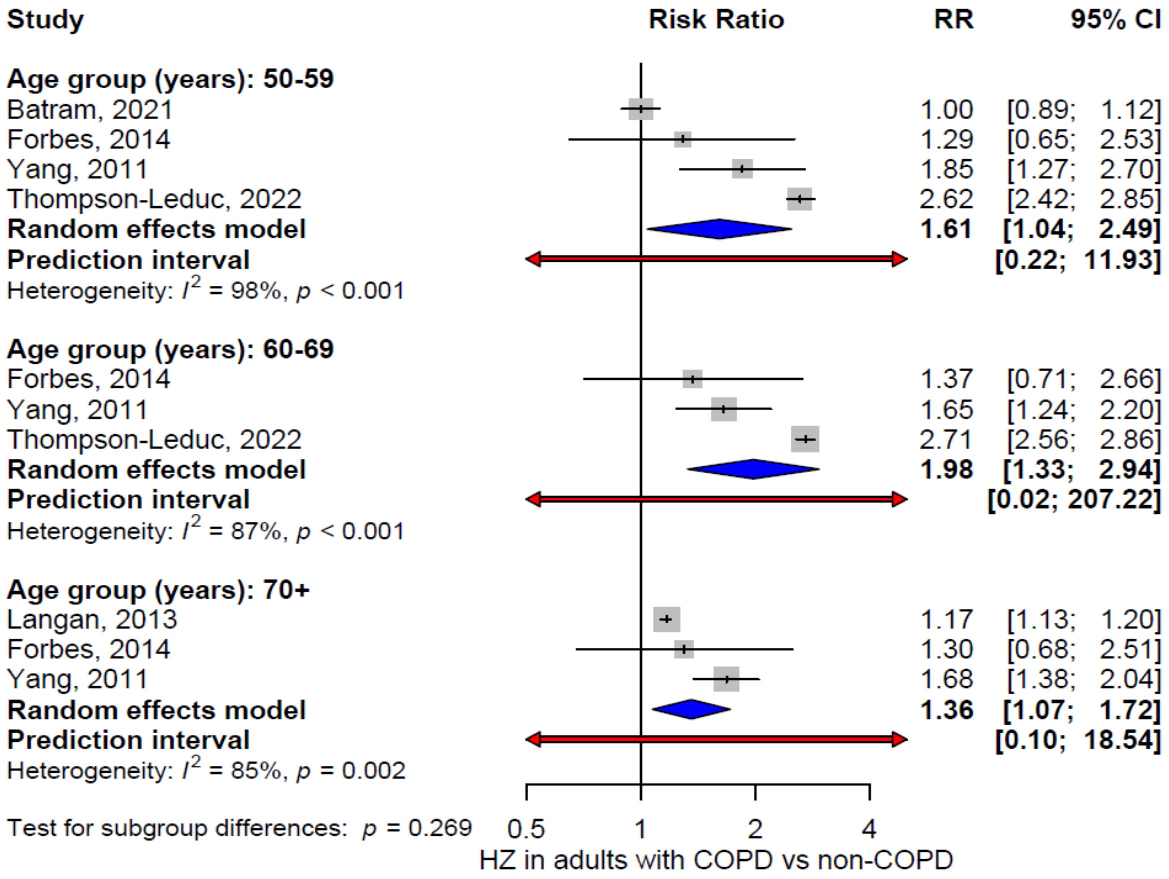

95% CI: 95% confidence interval; COPD: chronic obstructive pulmonary disease; HZ: herpes zoster; RR: risk ratio

Table S4. Healthcare resource use and costs

Author, year Country Period	Age group	In/ outpatient	Type of HCRU	Measure	Stratified by	Value	95% CI	Matched/adjusted by
**HCRU**								
Calabria 2020 [5] Italy 2013–2015	≥50y	Both	HZ-related hospitalisation	Proportion	NA	8.8	NR	Crude
			HZ-related LOS	Mean	NA	18.4 days	NR	
Gil-Prieto 2011 [20] Spain 1998–2004	≥50y	Inpatient	HZ-related LOS	Mean	50–59y	15.9 days	NR	Crude
					60–69y	14.2 days	NR	
					≥70y	12.9 days	NR	
Yawn, 2022 [17] US 2020	≥50y	Outpatient	HZ-related use of any type of healthcare services	Proportion	NA	98.30	NR	Crude
			HZ-related hospitalisation	Proportion	NA	10.90	NR	
Ghaswalla 2021* [6] US 2013–2018	≥50y	Both	Any medical service use	IR PPPY	All-cause	39.63	NR	Crude
					COPD-related	13.22		
			Inpatient medical service use	IR PPPY	All-cause	0.24	NR	
					COPD-related	0.18		
			ED medical service use	IR PPPY	All-cause	1.85	NR	
					COPD-related	1.00		
			Outpatient medical service use	IR PPPY	All-cause	29.57	NR	
					COPD-related	8.08		
			Other	IR PPPY	All-cause	7.97	NR	
					COPD-related	3.95		
			Any medical service use	IR PPPY	All-cause; 50–64y	36.05	NR	
					COPD-related	10.96		
			Inpatient medical service use	IR PPPY	All-cause; 50–64y	0.25	NR	
					COPD-related	0.18		
			ED medical service use	IR PPPY	All-cause; 50–64y	2.41	NR	
					COPD-related	1.19		
			Outpatient medical service use	IR PPPY	All-cause; 50–64y	26.95	NR	
					COPD-related	6.60		
			Other	IR PPPY	All-cause; 50–64y	6.43	NR	
					COPD-related	2.99		

			Any medical service use	IR PPPY	All-cause; 65–79y	38.62	NR	
					COPD-related	13.24		
			Inpatient medical service use	IR PPPY	All-cause; 65–79y	0.22	NR	
					COPD-related	0.17		
			ED medical service use	IR PPPY	All-cause; 65–79y	1.68	NR	
					COPD-related	0.96		
			Outpatient medical service use	IR PPPY	All-cause; 65–79y	28.86	NR	
					COPD-related	8.06		
			Other	IR PPPY	All-cause; 65–79y	7.86	NR	
					COPD-related	4.05		
			Any medical service use	IR PPPY	All-cause; ≥80y	44.34	NR	
					COPD-related	14.70		
			Inpatient medical service use	IR PPPY	All-cause; ≥80y	0.27	NR	
					COPD-related	0.20		
			ED medical service use	IR PPPY	All-cause; ≥80y	1.86	NR	
					COPD-related	0.98		
			Outpatient medical service use	IR PPPY	All-cause; ≥80y	32.94	NR	
					COPD-related	9.13		
			Other	IR PPPY	All-cause; ≥80y	9.27	NR	
					COPD-related	4.39		
**Ratio of HCRU for HZ in COPD vs. HZ in no COPD patients and pre-HZ vs. post-HZ HCRU analysis**								
Muñoz-Quiles 2018 [8] Spain 2009–2014	≥50y	Both	HZ-related outpatient visit	RR	NA	1.05	1.03–1.08	Crude
			HZ-related medication use	RR	NA	1.25	1.19–1.31	
			HZ-related hospitalisation	OR	NA	2.66	2.17–3.24	
			HZ-related sick leave	Mean ratio	NA	1.06	0.62–1.82	
			HZ-related outpatient visit	RR	ICS users	1.07	1.03–1.10	
			HZ-related medication use	RR	ICS users	1.45	1.32–1.59	
			HZ-related hospitalisation	OR	ICS users	2.59	1.80–3.62	
			HZ-related sick leave	Mean ratio	ICS users	1.06	0.34–3.29	
			HCRU 6 months post-HZ vs. 6 months pre-HZ	RR	Outpatient visits	1.02	0.96–1.08	
				OR	Hospitalisations	1.13	0.96–1.33	
				Mean ratio	Hospital LOS	1.19	1.00–1.42	
				RR	ICS use	0.98	0.76–1.25	
**Ratio of HCRU in COPD patients with HZ vs. COPD patients without HZ**								
	≥50y	Both	Any medical service use	aIRR	All-cause	1.17	1.14–1.21	

Ghaswalla 2021*[6] US 2013–2018					COPD-related	1.27	1.21–1.34	Adjusted for propensity score and relevant baseline characteristics (age, gender, geographic region, insurance type, comorbidities, immunosuppression status, use of oral or inhaled corticosteroids, and costs during the baseline period)
			Inpatient medical service use	aIRR	All-cause	0.92	0.82–1.04	
					COPD-related	1.00	0.88–1.13	
			ED medical service use	aIRR	All-cause	1.28	1.20–1.35	
					COPD-related	1.41	1.30–1.53	
			Outpatient medical service use	aIRR	All-cause	1.18	1.15–1.22	
					COPD-related	1.29	1.22–1.35	
			Other	aIRR	All-cause	1.13	1.07–1.19	
					COPD-related	1.26	1.13–1.41	
			Any medical service use	aIRR	All-cause; 50–64y	1.14	1.07–1.22	
					COPD-related	1.30	1.15–1.47	
			Inpatient medical service use	aIRR	All-cause; 50–64y	1.04	0.77–1.40	
					COPD-related	1.14	0.82–1.58	
			ED medical service use	aIRR	All-cause; 50–64y	1.27	1.10–1.47	
					COPD-related	1.64	1.35–1.99	
			Outpatient medical service use	aIRR	All-cause; 50–64y	1.18	1.10–1.27	
					COPD-related	1.37	1.21–1.54	
			Other	aIRR	All-cause; 50–64y	0.97	0.83–1.14	
					COPD-related	1.10	0.82–1.48	
			Any medical service use	aIRR	All-cause; 65–79y	1.15	1.11–1.20	
					COPD-related	1.24	1.16–1.32	
			Inpatient medical service use	aIRR	All-cause; 65–79y	0.88	0.75–1.04	
					COPD-related	0.94	0.79–1.11	
			ED medical service use	aIRR	All-cause; 65–79y	1.26	1.17–1.37	
					COPD-related	1.34	1.21–1.49	
			Outpatient medical service use	aIRR	All-cause; 65–79y	1.16	1.11–1.21	
					COPD-related	1.24	1.16–1.32	
			Other	aIRR	All-cause; 65–79y	1.12	1.05–1.21	
					COPD-related	1.26	1.10–1.45	
			Any medical service use	aIRR	All-cause; ≥80y	1.24	1.17–1.32	
					COPD-related	1.34	1.21–1.48	
			Inpatient medical service use	aIRR	All-cause; ≥80y	0.99	0.79–1.23	
					COPD-related	1.07	0.85–1.36	
			ED medical service use	aIRR	All-cause; ≥80y	1.34	1.20–1.49	
					COPD-related	1.41	1.21–1.64	

			Outpatient medical service use	aIRR	All-cause; ≥80y	1.25	1.17–1.32	
					COPD-related	1.35	1.22–1.50	
			Other	aIRR	All-cause; ≥80y	1.23	1.11–1.36	
					COPD-related	1.34	1.09–1.65	
**HZ costs**								
**Author, year Country Period**	**Age group**	**In/ outpatient**	**Cost measure**	**Perspective FU time**	**Stratified by**	**Cost 2023 €**	**SD/95% CI**	**Cost definition**
Calabria 2020 [5] Italy 2013–2015	≥50y	Both	Cost per hospitalised patient	NHS 1 year	NA	4 248	NR	Cost per hospitalised patient: primary diagnosis of HZ only Antiviral costs: brivudine, acyclovir, valacyclovir and famciclovir
			Cost per patient treated with antivirals		NA	127	NR	
Gil-Prieto 2011 [20] Spain 1998–2004	≥50y	Inpatient	Cost per hospitalisation	Hospital NR	50–59 y	6 111	NR	Cost per hospitalisation: primary or secondary diagnosis of HZ
					60–69y	5 430	NR	
					≥70y	5 448	NR	
Ghaswalla 2021 [6] US 2013–2018	≥50y	Both	Mean total all-cause cost	Payer 1 year	NA	3 769	6 933	All costs are PPPM All-cause: not defined COPD-related: a claim where a COPD diagnosis may be listed in any position or order HZ-related: a claim associated with an HZ diagnosis in any position Other costs: skilled nursing facilities, home care services, hospice, vision care, durable medical equipment, services and supplies,
			Mean inpatient all-cause cost		NA	720	NR	
			Mean ED patient all-cause cost		NA	884	NR	
			Mean outpatient all-cause cost		NA	1 536	NR	
			Mean other all-cause cost		NA	128	NR	
			Mean pharmacy all-cause cost		NA	501	NR	
			Mean total COPD-related cost		NA	1 403	3 623	
			Mean inpatient COPD-related cost		NA	301	NR	
			Mean ED patient COPD-related cost		NA	514	NR	
			Mean outpatient COPD-related cost		NA	384	NR	
			Mean other COPD-related cost		NA	63	NR	
			Mean pharmacy COPD-related cost		NA	140	NR	
			Mean total HZ-related cost		NA	222	961	
			Mean total medical services HZ-related cost		NA	218	961	
			Mean inpatient HZ-related cost		NA	56	466	
			Mean ED patient HZ-related cost		NA	96	642	

			Mean outpatient HZ-related cost		NA	56	481	and transportation services
			Mean other HZ-related cost		NA	10	124	
			Mean pharmacy HZ-related cost		NA	5	15	
			Mean total all-cause cost		50–64y	4 025	NR	Inpatient/ED/ outpatient/pharmacy costs not defined
			Mean total all-cause cost		65–79y	3 737	NR	
			Mean total all-cause cost		≥80y	3 668	NR	
			Mean total COPD-related cost		50–64y	1 277	NR	
			Mean total COPD-related cost		65–79y	1 365	NR	
			Mean total COPD-related cost		≥80y	1 576	NR	
			Adjusted total cost differences all-cause: COPD+/HZ+ vs. COPD+/HZ-		NA	285	100–488	
			Adjusted inpatient cost differences all-cause: COPD+/HZ+ vs. COPD+/HZ-		NA	-98	-174–12	
			Adjusted ED patient cost differences all-cause: COPD+/HZ+ vs. COPD+/HZ-		NA	105	48–165	
			Adjusted outpatient cost differences all-cause: COPD+/HZ+ vs. COPD+/HZ-		NA	174	91–260	
			Adjusted other cost differences all-cause: COPD+/HZ+ vs. COPD+/HZ-		NA	13	4–22	
			Adjusted pharmacy cost differences all-cause: COPD+/HZ+ vs. COPD+/HZ-		NA	26	-3–59	
			Adjusted total cost differences COPD-related: COPD+/HZ+ vs. COPD+/HZ-		NA	138	52–227	
			Adjusted inpatient total cost differences COPD-related: COPD+/HZ+ vs. COPD+/HZ-		NA	10	-38–53	
			Adjusted ED patient cost differences COPD-related: COPD+/HZ+ vs. COPD+/HZ-		NA	78	38–118	
			Adjusted outpatient cost differences COPD-related: COPD+/HZ+ vs. COPD+/HZ-		NA	70	41–99	
			Adjusted other cost differences COPD-related: COPD+/HZ+ vs. COPD+/HZ-		NA	15	8–20	

			Adjusted pharmacy differences COPD-related: COPD+/HZ+ vs. COPD+/HZ-		NA	-3	-9–4	
**PHN costs**								
Ghaswalla 2021 [6] US 2013–2018	≥50y	Both	Mean PHN-related cost of medical services	Payer 1 year	NA	51	438	All costs are PPPM PHN-related: a claim associated with a PHN diagnosis in any position
			Mean PHN-related cost of inpatient services		NA	13	204	
			Mean PHN-related cost of ED services		NA	23	327	
			Mean PHN-related cost of outpatient services		NA	13	127	
			Mean PHN-related cost of other services		NA	2	40	
**HZO costs**								
Ghaswalla 2021 [6] US 2013–2018	≥50y	Both	Mean HZO-related cost of medical services	Payer 1 year	NA	22	493	All costs are PPPM HZO-related: a claim associated with an HZO diagnosis in any position
			Mean HZO-related cost of inpatient services		NA	4	105	
			Mean HZO-related cost of ED services		NA	14	476	
			Mean HZO-related cost of outpatient services		NA	3	33	
			Mean HZO-related cost of other services		NA	2	35	

aIRR: adjusted incidence rate ratio; CI: confidence interval; COPD: chronic obstructive pulmonary disease; ED: emergency department; FU: follow up; HZ: herpes zoster; HZO: herpes zoster ophthalmicus; ICS: inhaled corticosteroids; IR: incidence rate; IRR: incidence rate ratio; LOS: length of stay; NA: not applicable; NHS: national health system; NR: not reported; OR: odds ratio; PHN: postherpetic neuralgia; PPPM: per person per month; PPRN: Patient-Powered Research Network; SD: standard deviation; RR: risk ratio; SIA: ambulatory information system; US: United States. *All-cause: not defined; COPD-related: a claim where a COPD diagnosis may be listed in any position or order; Inpatient/ED/ outpatient/pharmacy costs not defined; Other: skilled nursing facilities, home care services, hospice, vision care, durable medical equipment, services and supplies, and transportation services.

References

1. Imafuku S, Dormal G, Goto Y, et al. Risk of herpes zoster in the Japanese population with immunocompromising and chronic disease conditions: Results from a claims database cohort study, from 2005 to 2014. *J Dermatol* 2020: 47(3): 236-244. https://doi.org/10.1111/1346-8138.15214

2. Morena D, Lumbreras S, Rodríguez JM, et al. Chronic respiratory diseases as a risk factor for herpes zoster infection. *Archivos de bronconeumologia* 2023: 59(12): 797-804. https://doi.org/10.1016/j.arbres.2023.08.010

3. Langan SM, Smeeth L, Margolis DJ, et al. Herpes zoster vaccine effectiveness against incident herpes zoster and post-herpetic neuralgia in an older US population: a cohort study. *PLoS Med* 2013: 10(4): e1001420. https://doi.org/10.1371/journal.pmed.1001420

4. Batram M, Witte J, Schwarz M, et al. Burden of herpes zoster in adult patients with underlying conditions: Analysis of german claims data, 2007-2018. *Dermatol Ther (Heidelb)* 2021: 11(3): 1009-1026. https://doi.org/10.1007/s13555-021-00535-7

5. Calabria S, Ronconi G, Dondi L, et al. Patterns of prescription, hospitalizations and costs of herpes zoster in patients at risk, from a large Italian claims database. *Glob Reg Health Technol Assess* 2020: 7: 66-71. https://doi.org/10.33393/grhta.2020.2026

6. Ghaswalla P, Thompson-Leduc P, Cheng WY, et al. Increased health care resource utilization and costs associated with herpes zoster among patients aged >/=50 years with chronic obstructive pulmonary disease in the United States. *Chronic Obstr Pulm Dis* 2021: 8(4): 502-516. https://doi.org/10.15326/jcopdf.2021.0222

7. Imafuku S, Matsuki T, Mizukami A, et al. Burden of herpes zoster in the Japanese population with immunocompromised/chronic disease conditions: results from a cohort study claims database from 2005-2014. *Dermatol Ther (Heidelb)* 2019: 9(1): 117-133. https://doi.org/10.1007/s13555-018-0268-8

8. Munoz-Quiles C, Lopez-Lacort M, Diez-Domingo J. Risk and impact of herpes zoster among COPD patients: a population-based study, 2009-2014. *BMC Infect Dis* 2018: 18(1): 203. https://doi.org/10.1186/s12879-018-3121-x

9. Thompson-Leduc P, Ghaswalla P, Cheng WY, et al. Chronic obstructive pulmonary disease is associated with an increased risk of herpes zoster: A retrospective United States claims database analysis. *Clin Respir J* 2022: 16(12): 826-834. https://doi.org/10.1111/crj.13554

10. Yang YW, Chen YH, Wang KH, et al. Risk of herpes zoster among patients with chronic obstructive pulmonary disease: a population-based study. CMAJ : Canadian Medical Association journal = journal de l'Association medicale canadienne. 5 ed, Canada, 2011; pp. E275-280.

11. Forbes HJ, Bhaskaran K, Thomas SL, et al. Quantification of risk factors for herpes zoster: population based case-control study. *BMJ* 2014: 348: g2911. https://doi.org/10.1136/bmj.g2911

12. Joesoef RM, Harpaz R, Leung J, et al. Chronic medical conditions as risk factors for herpes zoster. *Mayo Clinic Proceedings* 2012: 87(10): 961-967. https://doi.org/10.1016/j.mayocp.2012.05.021

13. Kwon HJ, Bang DW, Kim EN, et al. Asthma as a risk factor for zoster in adults: A population-based case-control study. *The Journal of allergy and clinical immunology* 2016: 137(5): 1406-1412. https://doi.org/10.1016/j.jaci.2015.10.032

14. Lasserre A, Blaizeau F, Gorwood P, et al. Herpes zoster: family history and psychological stress-case-control study. *J Clin Virol* 2012: 55(2): 153-157. https://doi.org/10.1016/j.jcv.2012.06.020

15. Schmidt SAJ, Vestergaard M, Baggesen LM, et al. Prevaccination epidemiology of herpes zoster in Denmark: Quantification of occurrence and risk factors. *Vaccine* 2017: 35(42): 5589-5596. https://doi.org/10.1016/j.vaccine.2017.08.065

16. Queenan JA, Farahani P, Ehsani-Moghadam B, et al. The prevalence and risk for herpes zoster infection in adult patients with diabetes mellitus in the Canadian Primary Care Sentinel Surveillance Network. *Can J Diabetes* 2018: 42(5): 465-469. https://doi.org/10.1016/j.jcjd.2017.10.060

17. Yawn BP, Merrill DD, Martinez S, et al. Knowledge and attitudes concerning herpes zoster among people with COPD: An interventional survey study. *Vaccines (Basel)* 2022: 10(3). https://doi.org/10.3390/vaccines10030420

18. Esteban-Vasallo MD, Domínguez-Berjón MF, Gil-Prieto R, et al. Sociodemographic characteristics and chronic medical conditions as risk factors for herpes zoster: a population-based study from primary care in Madrid (Spain). *Hum Vaccin Immunother* 2014: 10(6): 1650-1660. https://doi.org/10.4161/hv.28620

19. Forbes HJ, Bhaskaran K, Thomas SL, et al. Quantification of risk factors for postherpetic neuralgia in herpes zoster patients: A cohort study. *Neurology* 2016: 87(1): 94-102. https://doi.org/10.1212/wnl.0000000000002808

20. Gil-Prieto R, San-Martín M, Álvaro-Meca A, et al. Herpes zoster hospitalizations of patients with chronic illnesses in Spain, 1998–2004. *Vacunas* 2011: 12(3): 95-101. https://doi.org/10.1016/s1576-9887(11)70013-9

21. Munoz-Quiles C, Lopez-Lacort M, Orrico-Sanchez A, et al. Impact of postherpetic neuralgia: A six year population-based analysis on people aged 50 years or older. *The Journal of infection* 2018: 77(2): 131-136. https://doi.org/10.1016/j.jinf.2018.04.004

22. Sodergren E, Mardberg K, Nishimwe M, et al. Incidence and burden of herpes zoster in Sweden: A regional population-based register study. *Infectious diseases and therapy* 2024: 13(1): 121-140. https://doi.org/10.1007/s40121-023-00902-1
