# Supplementary figures and images for: Global herpes zoster burden in adults with COPD: a systematic review and meta-analysis
Source: Eur Respir Rev. 2026 Feb 4;35(179):250167. doi: 10.1183/16000617.0167-2025 (PMC12883191; doi:10.1183/16000617.0167-2025)

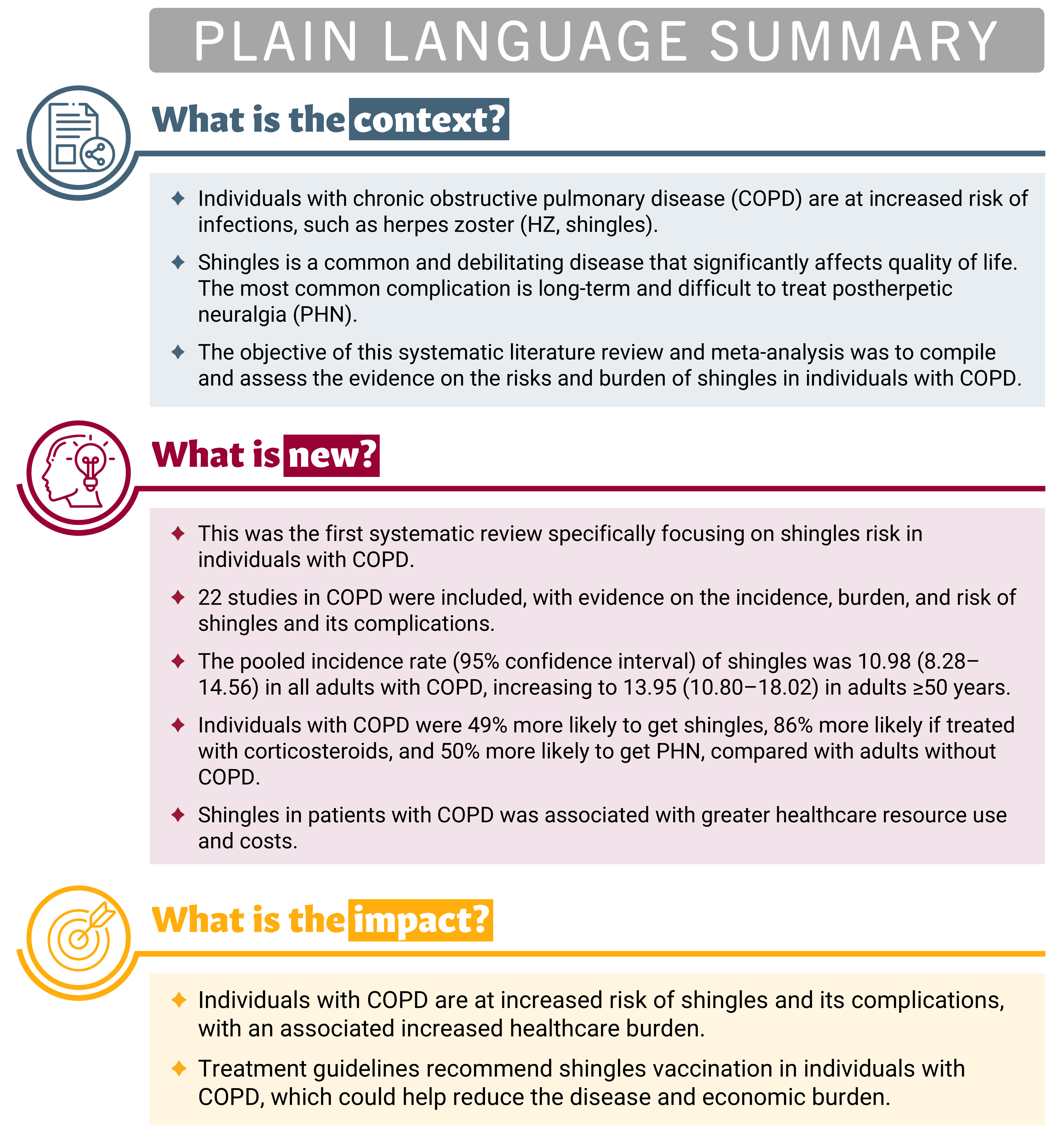

Supplement: Supplementary file 2 [file ERR-0167-2025.SUPPLEMENT.tif]
